# Notch Signaling Pathway in Cancer—Review with Bioinformatic Analysis

**DOI:** 10.3390/cancers13040768

**Published:** 2021-02-12

**Authors:** Dorota Anusewicz, Magdalena Orzechowska, Andrzej K. Bednarek

**Affiliations:** Department of Molecular Carcinogenesis, Medical University of Lodz, 90-752 Lodz, Poland; magdalena.orzechowska@umed.lodz.pl (M.O.); andrzej.bednarek@umed.lodz.pl (A.K.B.)

**Keywords:** Notch signaling, carcinogenesis, global signaling

## Abstract

**Simple Summary:**

The Notch signaling pathway, which controls multiple cell differentiation processes during the embryonic stage and adult life, is associated with carcinogenesis and disease progression. The aim of the present study was to highlight cancer heterogeneity with respect to the Notch pathway. Our analysis concerns the effects of the Notch signaling at different levels, including core components and downstream target genes. We also demonstrate overall and disease-free survival results, pointing out the characteristics of particular Notch components. Depending on tissue context, Notch members can be either oncogenic or suppressive. We observed different expression profile core components and target genes that could be associated with distinct survival of patients. Advances in our understanding of the Notch signaling in cancer are very promising for the development of new treatment strategies for the benefit of patients.

**Abstract:**

Notch signaling is an evolutionarily conserved pathway regulating normal embryonic development and homeostasis in a wide variety of tissues. It is also critically involved in carcinogenesis, as well as cancer progression. Activation of the Notch pathway members can be either oncogenic or suppressive, depending on tissue context. The present study is a comprehensive overview, extended with a bioinformatics analysis of TCGA cohorts, including breast, bladder, cervical, colon, kidney, lung, ovary, prostate and rectum carcinomas. We performed global expression profiling of the Notch pathway core components and downstream targets. For this purpose, we implemented the Uniform Manifold Approximation and Projection algorithm to reduce the dimensions. Furthermore, we determined the optimal cutpoint using Evaluate Cutpoint software to established disease-free and overall survival with respect to particular Notch members. Our results demonstrated separation between tumors and their corresponding normal tissue, as well as between tumors in general. The differentiation of the Notch pathway, at its various stages, in terms of expression and survival resulted in distinct profiles of biological processes such as proliferation, adhesion, apoptosis and epithelial to mesenchymal transition. In conclusion, whether oncogenic or suppressive, Notch signaling is proven to be associated with various types of malignancies, and thus may be of interest as a potential therapeutic target.

## 1. Introduction

Canonical Notch signaling is initiated by the interaction between a Notch ligand and Notch transmembrane receptor on the surface of a neighboring cell. In mammals, the Notch pathway consists of four equivalent receptors (NOTCH1-NOTCH4) and five ligands, including three Delta-like proteins (DLL-1, 2 and 4) and two Jagged proteins (Jagged-1 and Jagged-2) [1]. After the binding of the ligand to the Notch receptors at the cell surface, a two-step proteolysis cleavage process begins [2]. The first cleavage is catalyzed by the ADAM-family metalloproteases and leads to removal of the extracellular domain of the Notch, which is then targeted for lysosomal degradation. The residual part of the Notch protein, termed the Notch extracellular truncated (NEXT), undergoes a second cleavage mediated by gamma-secretase, an enzyme complex that contains presenilin, nicastrin, PEN2 and APH1. This causes the release of intracellular domain (NICD) from the transmembrane domain (TM). The NICD translocates into the nucleus and activates the transcription of the Notch target genes by forming an activator complex with DNA binding protein CSL and a member of mastermin-like (MAML) family of transcriptional co-activators [3,4]. The best characterized Notch targets are members of the HES and HEY gene families, which regulate aspects of the expression of genes involved in Notch-dependent cell-fate determination, such as apoptosis, proliferation or differentiation [4].

The evolutionarily conserved Notch signaling pathway controls various biological processes, including stem cell maintenance and adult tissue homeostasis [5]. In the following subsection, we will focus on the various role of the Notch pathway core members in different types of normal tissues. Then, based on the literature and our results, we will discuss how the activity of the Notch signaling pathway influences the carcinogenesis of different kinds of tumors. Finally, we will briefly describe cancer variability in terms of basic biological processes.

Notch signaling is essential for several steps of lung organogenesis [6]. Lung development occurs in five stages: the embryonic, pseudoglandular, canalicular, succular and alveorization. During the embryonic and pseudograndular stages, Notch ligands and receptors are expressed within proximal-distal airways, surrounding mesenchyme, which coordinates the proximal-distal patterning of branching morphogenesis, and endothelial cells. Expression of JAG1 and JAG2, particularly, is restricted to the distal tips, NOTCH2 and NOTCH3 are expressed in fetal lung mesenchyme, NOTCH3 and NOTCH4 in endothelial cells, while DLL1 expression is found in the proximal airways [7,8]. In addition to regulating early proximodistal cell fate, Notch is important for later differentiation of specific lineages, including secretory, ciliated, Club cells and neuroendocrine cell types [8]. NOTCH1 and NOTCH3, in response to JAG1, are involved in differentiation of airway epithelium into the secretory cells [9,10], whereas NOTCH2, with slight contribution from NOTCH1 and NOTCH3, mediates Clara/ciliated cell fate decision. In contrast, NOTCH1-NOTCH3 contribute in an additive manner and regulate the amount and size of neuroendocrine cells [11].

The kidneys are the body’s filters, but not only do they fulfill this excretory function, they also play an important role in maintaining the homeostasis of the internal environment of the body. The basic structural unit of the kidney is the nephron, which is made up of specialized epithelium cells. Several Notch signaling components are expressed throughout nephron development, including receptors NOTCH1/2, ligands JAG1 and DLL1, and targets genes HEY1 and HEYL, as well as modifiers of the Notch receptor/ligand affinity, such as LFNG [12,13,14]. Specifically, the Notch ligands JAG1 and DLL1 are expressed in renal vesicles (RV), and later are segregated to the middle of the S-shaped body. NOTCH1 and NOTCH2 are expressed throughout RV and S-shaped bodies, while NOTCH2 expression is also detectable in nephron progenitor cells. Additionally, NOTCH1-NOTCH3 expression, along with that of JAG1, DLL1 and HES1, has been observed within the collecting duct lineage [15,16,17].

The intestine is a highly complex organ that serves many crucial functions, including digestion and nutrient absorption, metabolism, barrier maintenance and immunity. Under normal circumstances, Notch pathway components play an important role in the developing intestine, maintaining the balance between proliferation and differentiation in the intestinal epithelium. Notch ligands and receptors, together with downstream components HES1, are expressed in the epithelial layer of gut. Expression of NOTCH1 is restricted to the intestinal crypt compartment [18], specifically proliferative zone located within the middle-third of the colonic crypt is abundant with NOTCH1 and JAG1 expression [19]. The expression of the Notch ligand DLL1 is limited to a small fraction of crypt progenitors and in differentiated goblet cells [20], whereas the expression of another ligand, JAG1, is restricted to the villi in the enteroendocrine cells or in the crypt [21].

Among epithelial cells within the prostatic epithelium, three major types can be distinguished: luminal, basal and neuroendocrine. They differ with respect to their location, morphology, function and expression of specific cytokeratins [22,23]. Studies have implicated the Notch pathway in the regulation of prostate morphogenesis, and expression of specific Notch signaling members has been found in epithelial cells [24,25,26,27]. Specifically, NOTCH1 is expressed in both basal and luminal layers, whereas JAG1 and DLK1 are restricted to luminal and basal cells, respectively [24]. Moreover, Belandia et al. demonstrated that HEY1, together with androgen receptor (AR), is expressed in luminal epithelial cells, and that HEY1 importantly acts as corepressor of AR [28].

The female reproductive system consists of internal and external organs (such as endometrium, cervix, ovaries and genitals) that build a very complex system designed to carry out several functions. The human endometrium is a type of tissue that undergoes cyclic regeneration along with the menstrual cycle. Some of the Notch members have been identified as differentially regulated throughout the menstrual cycle. Specifically, NOTCH1 has been localized in both the endometrial luminal and glandular epithelium, with the highest expression in the mid-secretory phase in the latter, whereas NOTCH3 was detected in the endometrial luminal epithelium in the proliferative phase. Among ligands, JAG1 and DLL1 were found in the glandular and luminal epithelium, with elevated levels in mid-secretory phase compared to proliferative phase. Interestingly, they found high expression of NUMB in the glandular epithelium of women with primary infertility, compared to normal receptive endometrium. Moreover, HES was moderately expressed in the glandular and luminal epithelium, with elevated levels in the secretory phase [29].

Ovaries are critical female reproductive organs, supporting the development of the oocytes. Both embryonic and postnatal ovarian development have been confirmed to be characterized by Notch signaling, which is especially essential for follicle assembly and growth, meiotic maturation, vasculogenesis of ovaries, and steroid hormone production. Importantly, among all Notch core members, NOTCH2, JAG1, JAG2 AND HES1, HEY2 were the most abundantly expressed within embryonic ovaries. During embryonic ovarian development, the most abundantly expressed receptor is NOTCH2, while the most abundant ligands are JAG1 and JAG2. On the other hand, during follicle growth, JAG1, HES1 and HEY2 are upregulated in preantral follicles in contrast to NOTCH1, NOTCH2, JAG2 and HES5, which are lowered. In addition, HEY1 expression is dependent of the size of the preantral follicle [30].

Furthermore, emerging evidence indicates that Notch signaling plays a critical role in the development and growth of mammary glands. Using murine models, Raafat et al. demonstrated the temporal activity of the Notch in the epithelial cells of mammary glands during normal development. Regarding receptors, NOTCH3 was the most abundant during all developmental stages, in contrast to NOTCH4, the expression of which was undetectable. Among other members of the Notch pathway, JAG1, DLL3 and HEY2 showed the highest expression during different stages of postnatal mammary gland development [31]. Study of human normal breast tissue showed that Notch activation leads to self-renewal of stem cells and affects lineage-specific differentiation of progenitor cells [32].

Taking into account the wide spectrum of the Notch pathway trail in normal cell development, it is not surprising that dysregulated Notch signaling is increasingly related to disease and cancer. It should also be emphasized that, depending on the tissue context, Notch can variously serve as an oncogene or a tumor suppressor [33,34,35]. Depending on the expression profiles in specific tumor types, the Notch signaling pathway can be involved in either cell survival or death, proliferation or apoptosis, activation or blockade of differentiation, and prevent invasion or induce metastasis [36].

## 2. Materials and Methods

### 2.1. Data Collection

The expression profile data (RNASeqV2, level 3, RSEM normalized, data status of 28 January 2018) of bladder urothelial carcinoma (BLCA), breast invasive carcinoma (BRCA), cervical squamous cell carcinoma and endocervical adenocarcinoma (CESC), colon adenocarcinoma (COAD), kidney chromophobe (KICH), kidney renal clear cell carcinoma (KIRC), kidney renal papillary cell carcinoma (KIRP), lung adenocarcinoma (LUAD), lung squamous cell carcinoma (LUSC), ovarian serous cystadenocarcinoma (OV), prostate adenocarcinoma (PRAD), rectum adenocarcinoma (READ), uterine corpus endometrial carcinoma (UCEC) with their corresponding clinical data were obtained from The Cancer Genome Atlas (TCGA) database through GDAC Firehose (https://gdac.broadinstitute.org/, accessed 13 January 2020). Patients with missing clinical or expression values were excluded from further analysis. Additionally, normal solid tissues matching each cancer type (excluded OV) were retrieved using TCGA Assembler R package [37]. In total, our analysis included 5253 cancerous samples (BLCA-408, BRCA-1080, CESC-304, COAD-297, KICH-66, KIRC-533, KIRP-289, LUAD-515, LUSC-499, OV-301, PRAD-497, READ-94, UCEC-370) and 501 matching normal samples (BLCA-19, BRCA-113, CESC-3, COAD-41, KICH-25, KIRC-72, KIRP-32, LUAD-59, LUSC-51, PRAD, 52, READ-10, UCEC-24). The list of core members of the Notch signaling pathway were chosen based on the KEGG database (hsa04330) and downloaded from MsigDB (KEGG_NOTCH_SIGNALING_PATHWAY) [38]. A total of 53 genes were included in our analysis. Furthermore, a list of Notch downstream target genes was identified through the Gene Transcription Regulation Database (GTRD), available online at http://gtrd.biouml.org/ [39]. We listed a total of 2949 targets of Notch-specific transcription factors of HES/HESY families.

### 2.2. Notch-Associated Global Profiling of Tumors and Normal Tissues

Partitioning of different type of cancers and their corresponding normal tissues was performed based on expression data using the monocle3 R package. Using the monocle3 package, we applied the Uniform Manifold Approximation and Projection (UMAP) function, which is a nonlinear dimensionality reduction technique for large transcriptomic data. Dimension reduction is the process of transforming the original data set into a data set with fewer dimensions, while retaining the information carried in the data. Principal Component Analysis (PCA) is the most popular algorithm for linear dimension reduction. Basically, it relies on projecting data in such a way that the variance of the data in the low-dimensional representation is maximized. In contrast to PCA, UMAP, which is method based on manifold learning techniques, is adapted to nonlinear data. UMAP captures both the local and global structures of the datasets and preserves the high-dimensional topology of data points in the low-dimensional space. In monocle, the data were preprocessed using the PCA method, followed by the reduction of dimensions using the UMAP method, leading to the extraction of only the meaningful structure of the given population, while filtering out confounding noise. The principles of the UMAP approach in the context of genomic data are very well described in Diaz-Papkovich et al., 2019 [40].

### 2.3. Heatmaps and logFC Calculation

Basic gene expression differences between cancerous and normal samples were calculated using logarithmized fold-change (logFC). Furthermore, the heatmaps for expression profiling of differentially expressed genes were generated using the R package gplots and the heatmap.2 function. Additionally, hierarchical clustering was performed using the complete agglomeration method and the Spearman distance metric.

### 2.4. Survival Analysis

Disease-free survival (DFS) and overall survival (OS) were determined using particular core member genes as biomarkers using the Evaluate Cutpoint application [41]. In brief, DFS/OS analysis was preceded by optimal cutpoint determination, which is defined as the cutpoint of the most significant split, enabling patients to be categorized according to favorable or unfavorable prognosis based on the expression of a particular gene. In our analysis, we used the cutp algorithms of cutpoint determination in correlation with survival time and clinical outcome according to the following clinical parameters: “patient.person_neoplasm_cancer_status” and “patient.vital_status” as event indicator and “patient.days_to_last_followup” and “patient.days_to_death” as time of observation for DFS and OS, respectively.

### 2.5. Mutations

In addition to the above-mentioned analysis, mutations and copy number alterations (CNA) occurring in pathway core genes were identified via cBioPortal [42] among the respective cohorts.

## 3. Results and Discussion

In the review above, we focused on recent studies linking normal Notch signaling with different tissues. To show the transcriptomic differences of a total of 53 Notch core members among various kinds of malignancies (BLCA, BRCA, COAD, CESC, KICH, KIRC, KIRP, LUAD, LUSC, OV, PRAD, READ and UCEC), we used TCGA data and performed a global analysis of expression profiles. We focused on the effects of the Notch core alterations in terms of a general comparison between cancer type, a comparison between cancerous vs. normal tissues, and finally, the clinical outcome of the crucial core Notch members, including ligands, receptors, modulators and transcription factors (overall survival (OS) and disease-free survival (DFS) analysis). By applying Uniform Manifold Approximation and Projection (UMAP), we investigated the heterogeneity between the previously mentioned cancer types and their corresponding normal tissues. This machine learning approach for dimension reduction in large transcriptomic data was preceded by principal component analysis (PCA). The DFS and OS analysis were performed based on the determination of expression cutpoint, enabling patients to be split into categories according to favorable or unfavorable prognosis with reference to their relative level of expression within cancer tissues, marking the oncogenic or suppressive character of a particular gene.

**Figure 1:**
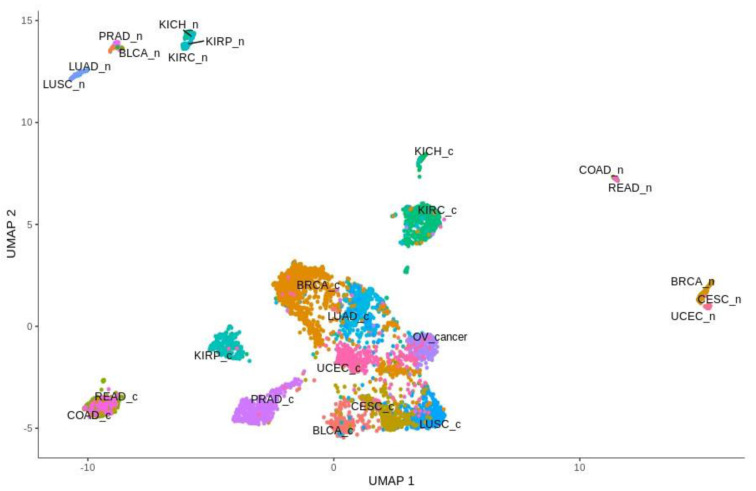
Spatial profiling of BLCA, BRCA, CESC, COAD, KICH, KIRC, KIRP, LUAD, LUSC, OV, PRAD, READ and UCEC accompanied by normal tissue samples with respect to expression of Notch core components. Tumors are designated with a “c” ending, while normal tissues are designated with an “n” ending.

**Figure 2:**
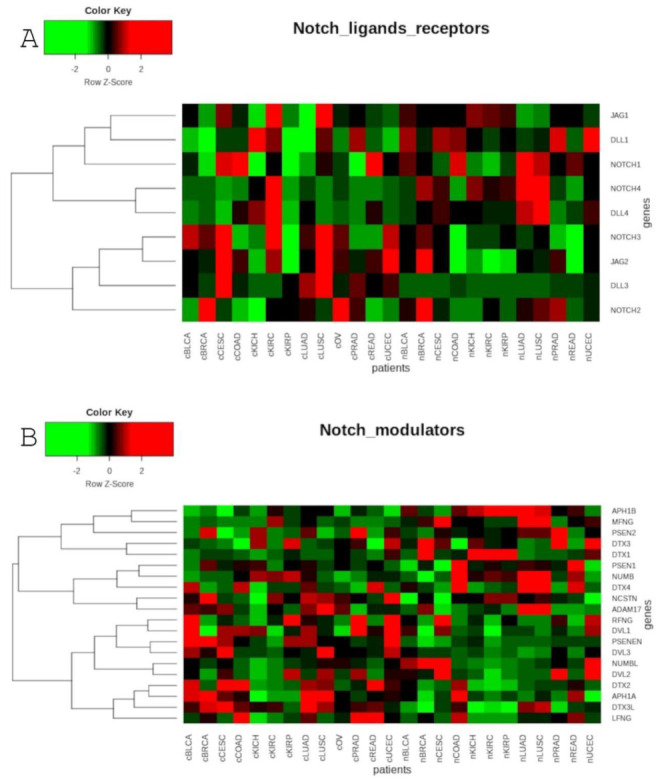
Heatmap reflecting the differential median expression of the Notch core components in the analyzed cancers and normal tissues: (**A**) ligands and receptors; (**B**) modulators.

**Figure 3:**
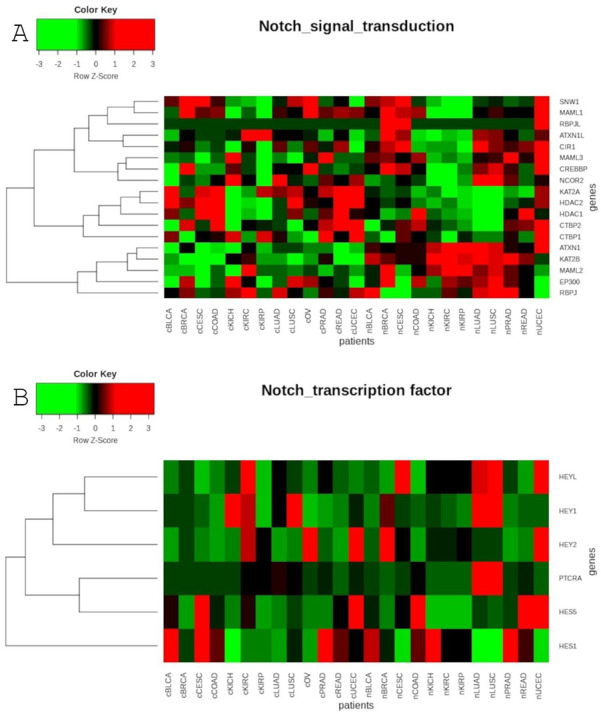
Heatmap reflecting the differential median expression of the Notch core components in the analyzed cancers and normal tissues: (**A**) signal transductors; (**B**) transcription factors.

The global analysis profiling expression of the Notch core components among patients with the above-mentioned cancers revealed a clear spatial portioning of each cancer type, and an even more visible portioning of normal samples within UMAP spaces (Figure 1). Although most of the tumors were clustered in one central cloud, it was clear that they were separated from each other. Only COAD and READ samples tended to be mixed together, forming one cluster that was considerably distant from the others. The most distinct tumors with respect to the Notch core were COAD and READ, mentioned previously, as well as all types of kidney carcinoma—KICH, KIRC and KIRP. Moreover, KIRP was significantly separated from KICH and KIRC in UMAP2, whereas KIRC and KICH seemed to be more similar to each other with both UMAP1 and UMAP2. Furthermore, PRAD appeared to be discrete from the central cluster, but to a lesser extent. Interestingly, LUAD and LUSC, which belonged to the central cluster, lay on the opposite side of this large group of samples with UMAP1, and LUAD seemed to be more similar to BRCA, whereas LUSC was more similar to CESC. It is worth noting that female hormonal cancers such as BRCA, OV and CESC also had places in the central cluster, but they were located on opposite sides of this cluster. Importantly, normal tissue was partitioned at the extreme opposite end of the UMAP spaces (Figure 1). The general results of UMAP are reflected in the expression profile of individual Notch members shown in Figure 2 and Figure 3.

### 3.1. Signaling of the Core Components—Ligands and Receptors

In light of the multiple and various roles that Notch signaling plays in the development of normal tissues, it is not surprising that abnormal expression of Notch pathway members is linked with multiple diseases and cancers. The ligands (canonical: DLL1, DLL3, DLL4, JAG1 and JAG2) and receptors (NOTCH1-4) are the most widely described members of the Notch pathway. Several independent studies have discovered that NOTCH1 overexpression is correlated with tumor progression, poor prognosis and proliferation of lung cancer cell lines [43,44,45]. Moreover, in vitro and in vivo data have shown that NOTCH1 activation by ADAM17 results in the tumorigenicity of NSCLC cells [46]. On the other hand, Wael et al. revealed that NOTCH1 has a suppressive effect on lung adenocarcinoma cell lines, but not on lung squamous cell carcinoma cell lines [47]. Consistent with this report, a study by Zhi-Yan Liu et al. suggested that Notch overexpression was associated with better outcomes in LUAD [48]. In the TCGA data, we observed that NOTCH1 expression was decreased approximately two-fold among LUAD patients in comparison with normal samples (Table 1). These results, along with the previous ones, could indicate positive activity of NOTCH1 in neoplastic tissues. Nonetheless, OS and DFS analysis did not show significant results with respect to LUAD, but revealed decreased NOTCH1 expression associated with a more favorable prognosis in LUSC (Table 2 and Table 3). Combining these findings, opposing roles of NOTCH1 in lung cancers could be suggested, with it being a suppressor in LUAD and an oncogene in LUSC. Moreover, there was a slight difference in NOTCH1 expression between LUAD and LUSC, but even larger differences in the expression of NOTCH3, JAG1, JAG2 and DLL3 (Figure 2A). The previously mentioned study of Zhi-Yan Liu and colleagues also presented results for other Notch pathway members, and so, in addition to NOTCH1, elevated mRNA expression of NOTCH2, JAG1 and DLL1 was also associated with favorable overall survival in LUAD, whereas higher expression of NOTCH3, JAG2 and DLL3 was associated with poor survival [48]. On the other hand, high expression of JAG1 promotes tumor cell invasion and metastasis and is associated with poor prognosis in squamous cell carcinoma, which, together with the above-mentioned study, suggests a dual role for JAG1 in NSCLC [49]. Nevertheless, our results showed that low expression of JAG1 was associated with better OS, both in LUAD and LUSC, while low expression of JAG2 was associated with better OS and DFS in LUAD only. Additionally, in the case of LUSC, JAG1, as well as JAG2, showed higher expression compared to normal tissue (Table 1), thus confirming the oncogenic nature of these ligands. Regarding receptors, lower expression of NOTCH2 and NOTCH4 was favorable for OS in LUSC, whereas lower expression of NOTCH4 was also favorable for DFS in LUSC (Table 2 and Table 3). Interestingly, a frequency of over 13.6% was observed for NOTCH2 CNVs in LUAD (Table 4). Expression of another receptor, NOTCH3, was higher in LUSC cancer samples compared to normal (Table 1). Our findings refer to the previously established oncogenic role of NOTCH3 in LUSC, as well as LUAD, which states that its overexpression is significantly associated with TNM stage and lymph node metastasis in both types of NSCLC [50]. Moreover, elevated expression of NOTCH3 may contribute to resistance to chemotherapy in lung cancer patients [51]. An assessment of the role of NOTCH3 in cell adhesion, EMT and motility revealed that it behaves as a tumor suppressor in SCLC, while it acts as tumor promotor in NSCLC [52].

With respect to colorectal cancer (CRC), high expression of NOTCH1 has been revealed to be negatively correlated with NOTCH2, and thus has diverse effects on patient survival. High expression of NOTCH1 is associated with poor overall survival, while high expression of NOTCH2 indicates better survival [53]. This trend is in line with our data, although only in the case of READ, where low expression of NOTCH1 together with high expression of NOTCH2 was better for OS (Table 3). Thus, NOTCH1 and NOTCH2 could prove to perform various functions in tumorigenesis and progression in CRC. Furthermore, elevated expression of NOTCH1 increases cell migration, affects the ability to form anchorage-independent colonies, and promotes stemness in cancer cells through the alteration of CD44, SLUG, SMAD-3, JAG1, HES1 and E-cadherin expression [54]. Higher expression of JAG1 is associated with poorer survival rate and increased risk of recurrence, due to promotion of the epithelial-to-mesenchymal transition and cell proliferation [55]. The experimental tumor model suggested that ADAM17 proteolytic cleavage of JAG1 from endothelial cells results in the activation of Notch signaling by soluble JAG1 and promotes the cancer stem cell phenotype [56]. In addition, it has been reported that JAG1 contributes to increased recurrence, poor outcome and chemoresistance of Kras-mutated colorectal cancer [57]. Our results suggest that lower expression of JAG1 is actually better for OS, although only in READ, and not in COAD, patients. In contrast, higher expression of both JAG1 and JAG2 are correlated with better disease-free survival in COAD (Table 2 and Table 3). In the colon, JAG2 expression level was enhanced in tumor samples compared to the normal crypt base and differentiated epithelium [58]. Increased expression of JAG2 was observed in colorectal tumors of ApcMin/+ mice and human CRC cell lines. Moreover, inhibition of JAG2 modulates the sensitivity of CRC cells to chemotherapeutic agents [59]. We also found elevated expression of JAG2 in cancer samples both in COAD and READ (Table 1). Serafin et al. observed that another Notch ligand, DLL4, contributes to upregulation of NOTCH3 in colorectal cancer samples, and that silencing of NOTCH3 led to a reduction in proliferation and the inhibition of tumor growth [60]. Furthermore, NOTCH3 overexpression has been linked to the aggressive malignant colorectal cancer cell phenotype [61], as well as to tumor recurrence after surgical CRC resection [62]. Our data indicate that the expression of NOTCH3 was about two times higher in cancer samples compared to normal in both COAD and READ (Table 1), although this was not observed in the case of DLL4. These results reaffirmed the oncogenic character of NOTCH3 during colorectal carcinogenesis, but not in the accompaniment of the DLL4 ligand. Interestingly, despite COAD and READ exhibiting a common expression profile for ligands and receptors, they reflected varying trends with respect to clinical outcome (Table 1). Notch receptors and ligands affect patients’ overall survival and disease-free survival differently depending on tumor subtype, and thus we conclude that they should be taken into account as a separate set of potential prognostic markers for COAD and READ.

Although previous study based on the mRNA data of the patient cohorts showed some of the Notch pathway components to be prognostic factors that were uniquely associated with each of these subtypes, while some were differentially expressed among all subtypes [63], there have been very few reports related to Notch in specific types of renal carcinoma. In the case of the Notch pathway in renal cancer, the most examined type is clear cell renal cell carcinoma, and little is known about Notch in papillary and chromophobe renal cell carcinomas. The first thing that stands out in our results is the differences in the ligand and receptor expression profiles among these three subtypes (Figure 2A). In this respect, KIRC is distinctly different, with most the expression of most genes being elevated compared to KICH and KIRP. In terms of the individual genes, NOTCH1 expression was positively correlated with clear cell renal cell carcinoma (CCRCC) carcinogenesis and progression. Indeed, Zhuang Z. and colleagues reported that NOTCH1 protein expression was exhibited in patients with higher TNM stage or Fuhrman grade or larger tumor size [64]. Moreover, NOTCH1 and JAG1 expression were significantly elevated in localized and metastatic CCRCC tumors [65,66], and they were also linked to reduced overall and disease-free survival [67]. In contrast, our data showed minor differences in NOTCH1 expression between KIRC and normal tissue, but a decrease of more than half in NOTCH1 expression in KIRP with respect to normal (Table 1). Interestingly, it was low expression that correlated with a better prognosis for KIRP patients (Table 3), while in KICH and KIRC, enhanced NOTCH1 expression was correlated with improved survival. The same trend could be observed in the cases of DLL4, JAG1, NOTCH3 and NOTCH4, suggesting a different mechanism for the clinical outcome of KIRP patients. It is worth noting that similar set of genes (DLL1, DLL3, DLL4, JAG1, NOTCH3 and NOTCH4) predicted better disease-free survival with low expression (Table 2). Elevated levels of DLL4 were correlated with worse overall survival in clear cell renal cell carcinoma, and likewise with distant metastasis [68], which can be attributed to the fact that stimulation of renal cell line caki-1 by DLL4 resulted in overexpression of MMP2 and MMP9 [69], which are metalloproteinases strongly implicated in the metastatic process. The same study also indicated that expression of DLL4, NOTCH1, NOTCH2, HEY1 and HEY2 was upregulated in renal carcinoma tissues. Our results confirmed elevated levels of DLL4 in KIRC in comparison to normal samples, while in KIRP DLL4, NOTCH1, NOTCH3 and NOTCH4, it was downregulated compared to normal samples (Table 1). Indeed, in the case of NOTCH1 and NOTCH4, Sun et al. observed increased expression in the cytoplasm of epithelial cells in renal tubules, including the proximal tubules and distal convoluted tubules of non-neoplastic tissues, simultaneous with greatly reduced expression in human renal cell carcinoma. Additionally, NOTCH1 levels were found to be negatively correlated with tumor stage [70]. Since clear cell and papillary RCC are derived from the epithelial cells of the proximal tubule [71], the above results might suggest that NOTCH1 and NOTCH4 might be related to early stages of tumorigenesis of the KIRP subtype.

Rampias et al. and Maraver et al. reported in parallel the suppressive role of the Notch pathway in urinary bladder cancer by demonstrating loss of function mutation in NOTCH1 and NOTCH2 [72] and genetic mutation in Notch pathway components, especially in NOTCH1, -2 and -3 [73]. Notch inactivation was shown to be correlated with lower expression of Hes1 in squamous bladder cancer cells, and in turn, the expression of Hes1 was positively correlated with CDH1, and negatively with VIM, suggesting that the loss of Notch activity favors the EMT process [72]. Furthermore, the tumor suppressive role of the Notch receptor has been confirmed, and it was demonstrated that expression of NOTCH1 and NOTCH2, but also DLL1 and JAG1, was diminished in bladder tissues [74]. Meanwhile, another group showed that NOTCH2 acts as an oncogene by promoting tumor growth, invasion and metastasis in vivo and in vitro. Furthermore, NOTCH2 overexpression has been confirmed to be correlated with worse prognosis in muscle-invasive bladder cancer [75]. Our data also showed that lowered NOTCH2 expression was better for overall survival (Table 3). In support of this, the repression of NOTCH2 by miR-758-3p, and its inhibitory effect on cell proliferation, migration and invasion, has been examined and confirmed [76]. Regarding receptors, NOTCH3 demonstrated a similar effect on OS as NOTCH2, whereas higher NOTCH4 had a beneficial effect for DFS (Table 2 and Table 3). Overexpression of NOTCH3 was also implicated in enhancing growth and chemoresistance in bladder cancer, as well as poor prognosis and short overall survival in patients. NOTCH3 knockdown in the bladder cancer cell lines T24 and J82 resulted in decreased proliferation of these cells in vitro and lower tumor progression in vivo [77]. Additionally, it also seems that the Notch pathway is involved in angiogenesis through DLL4, the overexpression of which, when present in the bladder tumor vasculature, has been shown to be correlated with VEGF, an important signaling protein involved in both vasculogenesis and angiogenesis [78]. Consistent with the above, we also identified the oncogenic effects of DLL4 on overall survival (OS), whereas in the case of DFS, high expression indicated more favorable prognosis (Table 2 and Table 3). On the other hand, we found the expression of DLL3 in cancerous tissues was more than double that in normal tissues, and lower expression predicted better disease-free survival in BLCA patients (Table 1 and Table 2). Our analysis indicated that the tumorigenic character of DLL3 was associated with its increased expression in BLCA, as well as with disease recurrence. This finding confirmed earlier research demonstrating the overexpression of DLL3 in small-cell bladder cancer, indicating that the efficacy of DLL3-targeting antibody drug-conjugate was superior to chemotherapy [79].

One of the most studied Notch members in the prostatic epithelium is NOTCH1, which defines progenitor cells, and the elimination of which can lead to impaired branching morphogenesis, growth and the differentiation of early postnatal prostate in vitro [80]. Consistent with this, NOTCH1, along with HEY1, was found to be significantly downregulated in prostate adenocarcinomas compared to normal tissues [27]. Our data also suggested that high expression of NOTCH1 and HEY1 indicates better OS prognosis in PRAD (Table 3). On the contrary, recent studies have shown that NOTCH1 acts more like an oncogene than a tumor suppressor. It has been noticed that NOTCH1-knockdown reduces invasion and proliferation of LNCaP cells [81] as well as the metastatic properties of castration-resistant prostate cancer cells by regulating the EMT markers E-cadherin and vimentin [82]. This latter claim was confirmed in another study demonstrating that NOTCH1 overexpression in PC-3 cells is associated with EMT markers and cancer stem cells phenotype [83]. Furthermore, it has been shown that, simultaneously with NOTCH1 overexpression, patients with high-grade metastatic adenocarcinoma exhibit elevated levels of JAG1 expression compared to low-grade and non-metastatic groups [84]. Interestingly, in contrast to the case for OS, lowered expression of NOTCH1 is more favorable for DFS. Likewise, NOTCH3 and NOTCH4 were enhanced in prostate cancer patients as well as in prostate cancer cell lines compared to the normal cell lines, respectively. Overexpression of NOTCH3 was observed to be associated with lymph node metastasis, higher Gleason grade, and invasiveness [85]. Furthermore, Danza et al. demonstrated that the activation of NOTCH3, which sustains the proliferation of PC cells, can be triggered by hypoxia by altering the membrane structure of these cells [86]. Similar to NOTCH1, NOTCH4 silencing increases the level of E-cadherin while inhibiting the expression of vimentin and N-cadherin [87]. Indeed, our results suggested that elevated expression of all Notch receptors and the ligands JAG1 and JAG2 could be unfavorable for disease-free survival (Table 2). Additionally, an inhibitory ligand of the Notch pathway, DLL3, was found to be aberrantly expressed in advanced prostate cancers, and its overexpression is correlated with poor overall survival [88]. In our study, DLL3 was the only one among the ligands and receptors that had no effect on DFS, and its expression was slightly higher in tumors compared to normal samples (Table 2 and Table 3).

The tumorigenic properties of cervical cancer cells have been found to be modulated by cross-talk between NOTCH1 and RhoC. Srivastava et al. observed co-expression of these two molecules in primary cervical carcinoma biospecimens, and, remarkably, inhibition of Notch with GSI or NOTCH1 KO resulted in downregulation of RhoC followed by a significant decrease in cell migration and invasion in vitro [89]. Greater levels of NOTCH1, together with JAG1 overexpression, were observed in cervical cancer in comparison to normal specimens, and were associated with cervical cancer invasion, lymph node metastasis and FIGO system (staging scheme developed by the International Federation of Gynecology and Obstetrics) [90]. Indeed, elevated levels of JAG1 seemed to be unfavorable for both OS and DFS, but no significant results were obtained for NOTCH1 (Table 2 and Table 3). On the other hand, it has been shown that elevated levels of Notch expression result in growth arrest of HPV-positive cervical cancer cells [91,92]. In addition, most invasive cervical cancer (ICC) samples exhibit lower NOTCH1 expression, predominantly observed in the cytoplasm, than cervical intraepithelial neoplasia (CIN) samples, where nuclear localization of NOTCH1 was identified.

Immunihistochemical staining of the basal layer across proliferative and secretory phases revealed significantly higher expression of NOTCH1, NOTCH3, JAG1 and DLL4 among endometrial carcinoma samples compared to normal endometrium, independently of layer or phase. High NOTCH1 expression was also significantly associated with deep myometrial invasion, vessel involvement and ovarian metastasis. Moreover, patients with high NOTCH1, NOTCH3, as well as a combination of both high NOTCH1 and high JAG1, exhibited poorer OS than those with carcinomas with low or double-negative expression [93]. Consistent with this, our results indicated that lowered expression of NOTCH1, NOTCH2 and NOTCH3 was associated with better overall survival (Table 3). Another IHC study focused on the alteration of expression of NOTCH1, NOTCH4 and JAG1 in normal endometrial samples of pre- and postmenopausal women in comparison with unmatched pathological samples including, i.a., endometrial carcinoma. This study showed an upregulation of NOTCH1 in hyperplasia and carcinomas compared to polyps, whereas NOTCH4 and JAG1 decreased dramatically with increasing histological grade [94]. On the other hand, a study at the RNA level, including the quantification of Notch receptors (NOTCH1-4), ligands (JAG1, JAG2, DLL1) and HES1, revealed a significant decrease in the expression of all analyzed genes in endometrial carcinoma compared to matched, adjacent non-tumor endometrium [95]. Moreover, at the protein level, NOTCH1, NOTCH4 and DLL1 were more likely to be downregulated in stage IB than in IA tumors [96].

In ovarian tumors, it has been shown that JAG1 and NOTCH3 interaction promotes cell proliferation and adhesion [97]. In agreement with this, expression profiling of the Notch in serous OV vs. benign tissues revealed elevated levels of NOTCH3, JAG1 and JAG2 mRNA, as well as correspondingly higher levels of NOTCH3 and JAG3 proteins. Furthermore, NOTCH3 was correlated with poor OS and resistance to chemotherapy, and at advanced stages of disease, lymph node and distant metastasis at the protein level [98]. IHC staining revealed the presence of NOTCH1 in 95% of serous OV; however, staining was also observed in matched benign and normal ovarian controls, but only at marginal percentages (8% and 6%, respectively) [99]. Another IHC-based analysis in turn reflected the overexpression of DLL4 in tumor and endothelium in over 70% of OV samples, and this was ultimately associated with worse OS in contrast to samples with low DLL4 [100]. On the other hand, opposing results were obtained in the analysis we performed. Elevated expression of DLL4 seemed to be more favorable for OS than lowered expression (Table 3). Analysis of the correlation between Notch receptors and prognosis of OV patients reported that NOTCH1 significantly differentiated progression-free survival (PFS) with respect to TP53 mutation status, and its overexpression was correlated with worse prognosis. Upregulation of NOTCH2 in ovarian cancer patients was significantly correlated with poorer PFS, especially in grade II. Conversely, high NOTCH3 expression was more favorable for PFS in all OV cases, whereas elevated expression of NOTCH4 was significantly correlated with more favorable OS in all OV cases [101].

Breast cancer progression, as well as worse OS and DFS, has been repeatedly correlated with Notch receptors and ligands. Overexpression of NOTCH1 contributes to progression and, moreover, transition from ductal carcinoma in situ to invasive forms of cancer [102,103]. Furthermore, elevated co-expression of NOTCH1 and JAG1 mRNA has been observed and shown to be associated with poor overall survival in invasive human breast cancer [104]. In our analysis, elevated NOTCH1 expression was unfavorable for OS and also for DFS (Table 2 and Table 3). In addition, there is evidence suggesting a role for Notch signaling in metastasis due to its contribution in the EMT process. Specifically, Leong et al. found that inhibition of JAG1-dependent Notch activity led to attenuation of SLUG expression and re-expression of E-cadherin, accompanied by a reduction in tumor growth and metastasis through HEYL inhibition [105]. An oncogenic role can also be assigned to NOTCH4, which is correlated with poor prognosis following anti-estrogen treatment, and inhibition of which reduced breast cancer stem cells [106]. Indeed, low expression of NOTCH4 is more favorable, but only for DFS. Based on our analysis, lowering of NOTCH4, but also of NOTCH3, was unfavorable for OS (Table 2 and Table 3). Additionally, frequencies of over 12% and 11% of NOTCH2 CNVs were observed in BRCA and OV, respectively (Table 4). As for mutations in BRCA and OV, the frequency is very low, as for the other tumor types analyzed and described above. Nevertheless, the high mutation rate of the Notch core members has been found in plenty of disorders, such as T-cell acute lymphoblastic leukemia (T-ALL) [107], kidney diseases [108,109], oral squamous cell carcinoma [110] and head and neck carcinomas squamous cell carcinoma [111].

### 3.2. Signaling of the Core Components—Modulators

Of the Notch signaling modulators, the Fringe and Numb families are the most widely described in various type of cancers. The ligand binding to the receptor is tightly regulated by Fringe glycosyl-transferases (MFNG, LFNG and RFNG). MFNG and LFNG reduce their affinity for Jagged through glycosylation of the Notch receptors, so the receptors become more responsive to Delta-like ligands instead [112]. Our study revealed that in most cancer types, with the exceptions of KIRC, KIRP and PRAD, MFNG was lowered in cancer compared to normal samples. Interestingly, in case of LUSC only, we observed that, simultaneously with the low level of MFNG, one of the DLL ligands—DLL4—was also decreased, while both Jagged ligands were enhanced (Table 1). Indeed, the anti-tumor activity of MFNG in lung cancer has been supported by the study of Yi et al. The suppressive function of MFNG negatively regulates NOTCH3 activity through JAG1, which is a known ligand for NOTCH3 receptor [113]. On the other hand, this situation was not observed in our study regarding LUAD. With respect to the Numb family, Kikuchi et al. demonstrated that NUMB has the opposite role in both types of NSCLC. In LUAD, specifically, this leads to inhibition of tumor proliferation, migration and invasion, whereas in LUSC, it activates these processes [114]. Contrary to this, our OS analysis showed that both LUAD and LUSC have common trends, wherein lower expression of NUMB is favored (Table 3). Nevertheless, in our analysis, NUMBL was an inhibitor that showed different trends in OS for LUAD in comparison to LUSC, being an oncogene in the case of LUAD and a suppressor in the case of LUSC (Table 3). The suppressive character of NUMBL was factually demonstrated by the inhibition of growth and proliferation, as well as promotion of apoptosis in highly metastatic 95D lung cancer cells [115]. The reduced level of NUMBL in both LUAD and LUSC compared to normal tissues may confirm that LUSC has a more aggressive nature than LUAD (Table 1).

Little is known about Notch modulators in kidney cancers. There is evidence of reduced NUMB expression in clear cell carcinoma, while its elevated expression level suppresses cell viability, growth, proliferation and invasive ability [116]. On the other had, we identified the suppressive effect of NUMB on overall survival in KIRP, but not in KICH or KIRC (Table 3). For most of the modulators, their effect on OS and DFS is described for the first time. Specifically, the genes in the gamma secretase complex have different survival profiles in KIRC and KIRP. For example, in both KIRC and KIRP, decreased expression of PSEN2, PSENEN and NCSTN is favorable for overall survival, whereas in the case of disease-free survival, upregulation of PSEN2 is more favorable in KIRP. Elevated expression of PSEN1 was shown to offer better prognosis on OS and DFS in KIRC, only. Although elevated expression of APH1B is more favorable for OS in KIRC and KIRP, downregulation of APH1A is better for OS in KIRP (Table 2 and Table 3).

Similarly to lung cancer, the negative correlation between MFNG and JAG1 has also been shown in colorectal cancer. The absence of MFNG with a simultaneous high level of JAG1 was a predictor of poor prognosis in colorectal patients [117]. Our study showed that MFNG is indeed decreased in COAD and READ, but with a simultaneous elevation of JAG2, although not JAG1 (Table 1). Interestingly, lower expression of MFNG proved to be better for DFS in COAD. Moreover, most of the analyzed modulators showed similar tendencies to that of MFNG (Table 2). Overexpression of NUMBL triggers a decrease in colon cancer cell colony growth through the activation of the Notch pathway, although with a low-level decrease in sensitivity to chemotherapy and a correlation with poor prognosis in colon tumors [118]. In our data, NUMBL showed no significant effect on OS or DFS, but it was found that low expression of the NUMB paralog is associated with a better prognosis for DFS in COAD (Table 2 and Table 3).

So far, LFNG has been suggested as tumor suppressor in human prostate cancer cells. LFNG may inhibit Jagged-mediated NOTCH1/NOTCH4 signaling in the basal compartment and facilitate NOTCH3 signaling in luminal cells, consequently suppressing tumor initiation by preventing basal cell expansion and prostatic intraepithelial neoplasia formation [119]. Our data indicated that overexpression of LFNG as well as RFNG predicted better disease-free survival, whereas MFNG showed a distinctive trend. Moreover, elevated expression of gamma-secretase complex members PSEN2, PSENEN and NCSTN showed a favorable prognosis of DFS, whereas other modulators ADAM17, APH1A and NUMBL were favorable when lowered (Table 2). There is evidence suggesting that overexpression of ADAM17 contributes to prostate cancer cell proliferation through EGFR/PI3K/AKT pathway activation [120].

In vitro experiments on the reduction of NUMB expression in breast cancer cells resulted in growth suppression [121]. On the other hand, a close homolog of NUMB–NUMBL was shown to act as a tumor suppressor, and its absence may induce chemoresistance in tumor cells [118]. Consistent with this, we showed that elevated expression of NUMBL was significantly better for OS and DFS (Table 2 and Table 3). In cervical cancer, decreased NOTCH1 expression was found to be correlated with an increase in NUMB expression in ICC, as compared to CIN [122]. Furthermore, two splicing variants of NUMB (NUMB-L and NUMB-S) showed different consequences in cervical cancer. Specifically, elevated expression of NUMB-L led to HES1 and overexpression of HEY1 and proliferation of HeLa in vitro, and these effects were the contrary for the NUMB-S variant, which decreased them [123]. According to our results, downregulation of NUMBL and HES1 indicated a better prognosis with respect to DFS (Table 3).

### 3.3. Signaling of the Core Component—Signal Transductors and the Family of HES/HEY Transcription Factors

After engagement of the Notch receptor, proteolytic cleavage events release the intracellular domain of the Notch (NICD), which migrates to the nucleus, interacts with RBPJ, and recruits a coactivator complex composed of Mastermind (MAML). Reduced expression of RBPJ was observed in non-small cell lung cancers compared with normal bronchial epithelium from healthy individuals. Loss of RBPJ copy number occurred more frequently in lung squamous cell carcinoma compared with adenocarcinomas. In the same study, the human breast cancer model systems exhibited depletion of RBPJ, resulting in increased cell survival and enhanced tumorigenicity due to the signal relegation to MYC and NFκB [124]. In the present study, we did not notice any significant difference in expression level of RBPJ and RBPJL between tumor and normal tissues (Table 1). Nevertheless, as shown in Table 1 and Table 2, RBPJ, RBPJL, as well as MAML family members, had differentiated patient outcomes that reflected the oncogenic or suppressive characteristics of specific genes. MAML family transcriptional co-activators are integral regulators of Notch pathway activity. However, in the context of colorectal carcinoma, the link between the MAML1 and Wnt pathways has been revealed. Specifically, MAML1 has been determined to be a co-activator of transcription mediated by beta-catenin. Its knockdown in SW480 colon cancer cells affects the beta-catenin-induced expression of cyclin D1 and c-MYC, leading to tumor cell death [125]. On the other hand, in embryonic kidney cell MAML1, it increases the transcriptional activity of EGR1 and p300 promoters. Furthermore, bioinformatics analysis has revealed the association between p300, EGR1 and MAML1 gene alterations and increased overall survival in renal clear cell carcinoma [126]. In the study of breast cancer cell lines, MCF7 and MDA_MB-231, MAML1 has been demonstrated to be negative regulator of EMT markers expression [127]. These findings show that MAML1 participates in multiple signaling pathways and could have a pivotal role as a co-activator in signaling cross talk. Nevertheless, the relevance of other regulators has not been elucidated with respect to the discussed cancer types. Of the remaining regulators, KAT2A upregulation and positive correlation with tumor size has been reported in human non-small cell lung carcinoma [128]. We also observed double elevated expression of KAT2A in LUAD and LUSC, as well as in KIRP, BLCA, COAD and READ. Indeed, it has been demonstrated that KAT2A is overexpressed in human colon cancer, and this upregulation is induced by c-MYC and E2F1 [129]. On the other hand, we noticed that KAT2B is downregulated in most cancers compared to normal tissues (Table 1), indicating the characteristics of these two lysine acetyltransferases (KATs). While they have overlapping functions, KAT2A and KAT2B seem to be mutually exclusive in many types of cancer.

As already mentioned, the oncogenic role of NOTCH3 in human lung cancer has been described previously. Furthermore, it has been revealed that in both NSCLC and SCLC cells, expression of JAG1 and HES1 is affected by NOTCH3, suggesting that these proteins could be related to NOTCH3. Decreased expression of JAG1 and HES1 has been detected in cells with knock-down of NOTCH3, while they exhibit increased expression in H1688 cells transfected with N3ICD plasmid [52]. In addition, activation of HES1 in NSCLC is associated with tumor progression and tumor cell growth [130]. Consistent with this, we found that expression of HES1 was two times greater in LUSC than in normal tissues (Table 1). On the other hand, in LUAD, we observed lowered expression of HEY1, along with lowered expression of NOTCH1 and NOTCH4 compared to normal samples. A similar situation was found in KIRP, while in the case of KIRC, we observed heightened expression of HES5 and HEY1, at levels that were double those than in normal samples, in addition to heightened expression of NOTCH3 and NOTCH4 (Table 1). Moreover, HEY1 differentiated KIRC and KIRP regarding OS (Table 3). Interestingly, Liu and colleagues reported that the Notch pathway was involved in the progression of renal cancer influenced by long non-coding RNA. Both protein and mRNA levels of HES5 and HEY1 were downregulated in RCC cells with RP11-567G11.1 knockdown [131]. Other studies have suggested that upon NOTCH4 induction, HEY promotes the melanoma mesenchymal-to-epithelial transition (MET), and is important in promoting metastatic colonization [132]. Based on this information, more experimental evidence is necessary on NOTCH pathway in KIRC. Guo Z. et al. suggested that there was a strong positive relationship between ADAM17, NOTCH1 and HES1 in renal carcinoma by demonstrating that ADAM17 inhibition downregulated expression of NOTCH1 and HES1 more effectively that blockade of gamma-secretase [133].

In colorectal cancer, upregulation of HES1 has been found to be significantly correlated with distal metastasis at diagnosis and is an unfavorable prognostic factor for outcomes in colorectal cancer patients [134]. HES1 upregulates stem cell markers at the transcriptional level, and thus induces stem-like properties in colon cancer cells [135]. Moreover, HES1 has been found to increase MMP14 expression in CRC cells and promote cell invasion [136]. Along with HES1, overexpression of HEY1 is also correlated with worse CRC outcome [137]. Our data confirmed the oncogenic effect of HES1, as well as HES5 and HEY1, on OS, but only in READ patients, and not in COAD (Table 3).

Notch inactivation was shown to be correlated with lower HES1 expression in squamous bladder cancer cells, and in turn, expression of HES1 was positively correlated with CDH1, and negatively correlated with VIM, suggesting that the loss of Notch activity favors the EMT process [72]. Indeed, elevated expression of HES1 indicated better overall survival of BLCA patients. Similarly, HES1, along with HEY1, has been found to be favorable for PRAD overall survival when lowered (Table 3). HEY1 has been proven to be a co-repressor of activation domain (AF1) in the androgen receptor, and thus inhibits the transcription from androgen-dependent target genes. Repression of AR activity upon activation of Notch signaling could suggest a direct link between the endocrine pathway and the Notch pathway [28].

To date, alterations of the Notch-HEY1 axis have commonly been reported in breast cancer studies in vitro. Specifically, disruption of NOTCH-HEY1 signaling led to reduction in migration and invasiveness in association with downregulation of Hey proteins [138]. With respect to the Hes family, NOTCH-HES1 was shown to maintain BC stem cells [139], and elevated levels of HES1 were additionally related to the formation of bone metastasis through interactions with Runt-related protein 2 (Runx2) [140]. Additionally, the overexpression of HES1, as well as HES5, was found among cervical cancer cases, compared to CIN or normal cervical epithelia, and was furthermore correlated with poor prognosis of early-stage CESC patients [141]. These results were consistent with ours, which showed expression of HES1 and HES5 to be more than two times higher in CESC than in normal samples (Table 1).

### 3.4. Signaling of Notch Target Genes

After analyzing the core Notch pathway, we decided to focus on downstream signaling through Hes and Hey transcription factors. From the 2949 Hes and Hey targets, downloaded from GTRD database, we extracted the most essential groups, including apoptosis, adhesion and proliferation, as they are part of the principle process of cell fate determination, which is orchestrated mainly by Notch. Furthermore, we added 144 epithelial-to-mesenchymal markers, as this is a crucial process involved in Notch-mediated pathological situations.

**Figure 4:**
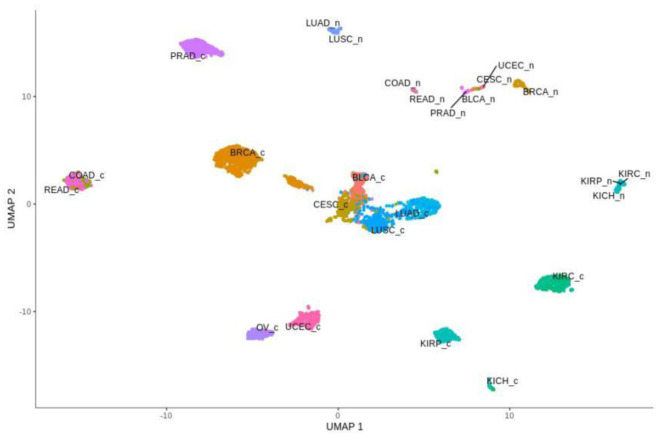
Spatial profiling of BLCA, BRCA, CESC, COAD, KICH, KIRC, KIRP, LUAD, LUSC, OV, PRAD, READ and UCEC, accompanied by normal tissue samples, with respect to expression of the Notch target genes. Tumors are designated with “c” endings, while normal tissues are designated with “n” endings.

Global profiling expression of the total of 3093 Notch targets and EMT markers among all of the above-mentioned cancers and their corresponding normal tissues revealed clear spatial partitioning within UMAP spaces (Figure 4). Unlike the Notch core, in this case, most of the tumors were not clustered in one main cloud, but rather they were clearly separated. Only two clusters were visible: the first consisted of BLCA, CESC, LUAD and LUSC, and the second was formed by COAD and READ. Other tumors were separated from these clusters and from each other to a greater or lesser extent. The most distinct cancers, both in terms of UMAP1 and UMAP2, were PRAD and KIRC, KIRP and KICH. Renal cancers were also on the opposite side to COAD and READ in UMAP1. It is worth mentioning that kidney cancers (KIRC, KIRP and KICH), despite their proximity, were still visibly separated, and interestingly, KIRC were located in close proximity to kidney normal tissues. Furthermore, the female hormonal cancers OV and UCEC were close to each other, but distant from BRCA, which formed an individual group. Importantly, normal tissue was partitioned toward the opposite end of the UMAP space. Most distinct from one another were groups of normal KIRC, KIRP and KICH tissues and groups of LUAD and LUSC.

**Figure 5:**
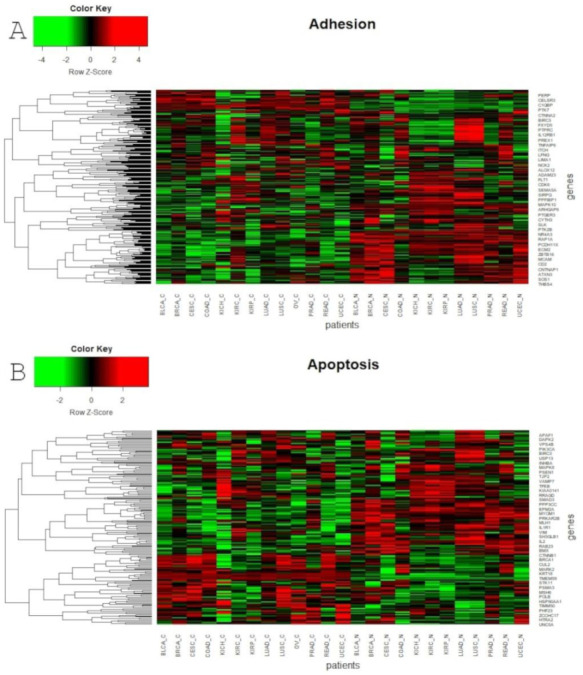
Heatmap reflecting differential median gene expression of Notch target genes in the analyzed cancerous and normal tissues. Target genes are grouped into four biological processes: (**A**) Adhesion; (**B**) Apoptosis.

**Figure 6:**
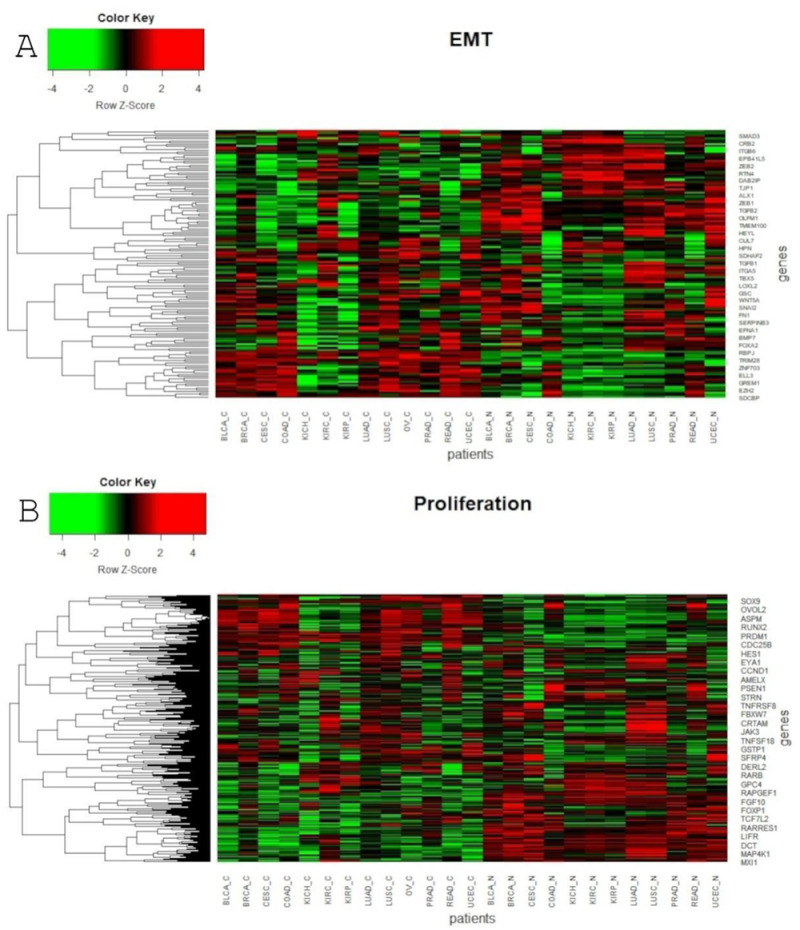
Heatmap reflecting differential median gene expression of Notch target genes in the analyzed cancerous and normal tissues. Target genes are grouped into four biological processes: (**A**) EMT; (**B**) Proliferation.

Global profiling using the UMAP approach was visualized using heatmaps (Figure 5 and Figure 6), which made it possible to distinguish tumor and corresponding normal tissues according to their ontological groups. The differences between the analyzed tumor and normal samples can easily be seen in every heatmap, especially with respect to adhesion and proliferation (Figure 5A and Figure 6B). Uncontrolled cellular proliferation is one of the most fundamental hallmarks of neoplastic cell populations [142]. Aberrant activation of multiple signaling pathways, for example Notch, Wnt, PI3K/Akt, and NF-kB, contributes to uncontrolled proliferation, and therefore carcinogenesis [143]. The Notch pathway plays a key role in cell proliferation through diverse mechanisms, including epithelial to mesenchymal transition (EMT). It is not surprising, therefore, that Notch targets belonging to the group of mesenchymal transition genes were differentially expressed between tumor and normal samples (Figure 6A). It is worth highlighting that kidney cancer types KIRC, KICH and KIRP apparently had different expression profiles than other cancers with respect to EMT and proliferation, and furthermore, there were also visible dissimilarities between KIRC and two others subtypes, KICH and KIRP (Figure 6A,B). Consistent with this, Chen et al. noted that the epithelial-to-mesenchymal transition pathway exhibited differential activity between renal cell cancer subtypes [144]. Conversion of epithelial cells into motile mesenchymal cells is carried out through the activation of EMT transcription factors (TFs), such as TWIST, SNAIL, SLUG and ZEB [145]. Importantly, the core EMT TFs are often co-expressed in various combinations to coordinate complex EMT programs, depending on the biological context. Activation of specific TFs may play a central role in EMT initiation in various biological states or tissue types [146,147]. Interestingly, among all of the cancer types, only KIRC showed elevated expression of ZEB1 and ZEB2 compared to normal samples, whereas SNAIL1, as well as KIRC, was also upregulated in COAD and READ (Figure 6A). Moreover, concordant with previous analysis, COAD and READ had almost identical expression profiles with respect to all ontologies (Figure 5 and Figure 6). Another example of similarities in expression profiles might be BLCA and CESC. Remarkably, although LUAD and LUSC were spatially distant with respect to Notch core genes, at the downstream level of targets, the difference was blurred (Figure 4). However, heatmaps showed slight differences in terms of increased NOTCH-dependent gene expression in the case of LUSC (Figure 5 and Figure 6). The dissimilarities between non-small cell lung carcinoma subtypes have been extensively described in the literature, indicating distinct tumor progression pathways [148,149]. These results suggest completely different mechanisms of recurrence in these two lung cancer subtypes.

## 4. Conclusions

All data presented here support the opinion that Notch signaling changes play a very important role in several aspects of carcinogenesis. Investigation of the Notch core and target gene expression patterns in various types of cancers resulted in the deep exploration of biological differences. Both the core members of the Notch pathway and its targets showed gene expression differentiation between tumors and significantly visible diversity between tumors and normal tissue. Particular differences between described cancer types can be seen in the comparison of neoplastic and normal tissues, especially for expression of the Notch ligands and receptors. The absence or low frequency of mutations and CNV in core Notch members may indicate that they have negligible impact for differentiation of Notch signaling between different cancers. The results confirmed previous reports that Notch expression activation depends on tissue context, inhibiting transformation in some tissues and promoting malignancy in others. Our analysis of patient survival showed that Notch deregulation resulted in similar or opposite tendencies for OS and DFS. An important observation is that Notch signaling differentiates between subtypes of the same tumor, which is very clearly visible in renal carcinomas (KIRC, KIRP and KICH), as well as in lung tumors (LUAD and LUSC). Interestingly, COAD and READ have almost identical expression profiles for the Notch core components, but in terms of OS and DFS, they are significantly distinct. These results suggest that Notch activity is responsible for biological differentiation resulting in distinct survival and recurrence programs of COAD and READ, although in terms of many other features, these tumors are practically identical. Furthermore, the diversity in successive steps of Notch signaling influenced the expression profile of target genes, as reflected in the various activity of genes involved in basic cellular processes such as proliferation, apoptosis, adhesion or EMT. All this provides new pathways for the development of targeted therapy. Depending on the tissue, points of therapeutic intervention may include ligands, receptors, regulators and transcription factors.

## Figures and Tables

**Table 1:** LogFC values of core Notch components. logFC was calculated between cancer and normal samples. Green color indicates double or higher expression in normal samples; red color indicates double or higher expression in cancer samples.

logFC												
GENE	BLCA	BRCA	CESC	COAD	KICH	KIRC	KIRP	LUAD	LUSC	PRAD	READ	UCEC
**Ligands and Receptors**												
DLL1	−1.354	−1.374	−0.703	−0.69	1.01	0.719	−2.996	−1.078	0.52	−0.159	−0.114	−1.755
DLL3	2.916	1.131	0	1.045	0	0	2.914	0	5.618	2.179	1.46	1.209
DLL4	−0.393	−0.82	−2.5	0.278	0.718	2.391	−1.844	−0.844	−2.157	−0.149	0.838	−1.239
JAG1	−0.246	−0.741	0.383	−0.105	−1.728	0.651	−0.803	−0.844	2.06	−0.009	−0.184	−0.069
JAG2	0.093	−0.948	0.662	1.661	−0.114	1.875	−0.259	0.118	1.261	−0.426	1.635	0.666
NOTCH1	−0.412	−0.813	0.83	0.208	−0.657	0.655	−1.086	−1.107	−0.343	−0.916	0.585	0.034
NOTCH2	−0.954	−0.463	−0.182	−0.325	−2.625	−0.023	0.482	−0.005	−0.662	−0.177	−0.509	−0.381
NOTCH3	0.732	−0.025	0.815	1.27	−0.287	1.392	−3.029	0.734	1.31	0.142	1.332	0.547
NOTCH4	−0.56	−1.67	−2.461	0.57	−0.678	1.63	−2.32	−1.986	−2.687	−0.535	0.835	−1.224
**Modulators**												
ADAM17	0.808	−0.211	1.653	0.369	−0.737	0.336	0.129	−0.41	0.411	−0.229	0.557	0.524
APH1A	0.255	0.785	0.239	−0.257	−0.293	−0.036	−0.124	0.465	0.433	0.056	−0.18	0.521
APH1B	−0.140	−0.189	0.289	−0.059	0.224	−0.116	−0.172	−0.303	−0.405	−0.008	−0.126	−0.421
DTX1	−0.561	−2.309	−0.09	−0.75	−4.817	−2.634	−1.646	0.164	0.025	0.463	−0.737	−0.247
DTX2	0.837	0.561	1.755	0.597	0.52	1.275	1.077	1.208	1.033	0.075	0.992	1.014
DTX3L	1.545	2.122	1.028	3.299	1.041	1.629	0.801	2.415	2.205	1.087	2.284	0.007
DTX3	−0.921	−1.443	−0.937	−3.819	0.587	−1.007	0.649	−2.103	−2.003	−0.782	−3.295	-0.221
DTX4	0.585	−0.891	0.827	−0.721	−2.85	0.048	−0.17	−0.572	−1.549	−0.383	−0.002	0.04
DVL1	0.396	0.234	0.069	0.515	0.413	0.32	0.043	0.466	0.183	0.697	0.421	0.175
DVL2	−0.038	−0.241	−0.742	−0.09	−0.316	0.275	0.678	0.107	0.583	−0.204	−0.064	−0.525
DVL3	0.416	0.379	0.74	0.149	−0.04	0.132	0.237	0.408	1.667	0.037	0.215	0.397
LFNG	0.086	0.384	−0.043	0.739	−1.956	0.511	0.95	0.805	−0.736	1.146	0.988	0.426
MFNG	−1.158	−1.481	−2.378	−1.276	−1.164	1.255	−0.104	−1.739	−2.538	−0.269	−1.273	−1.312
NCSTN	0.632	0.425	0.545	−0.169	−0.559	−0.001	−0.196	0.231	0.097	0.056	−0.027	0.942
NUMBL	−1.397	−2.373	−1.735	−3.364	−4.756	−3.497	−2.621	−2.703	−2.04	−1.842	−3.25	−1.423
NUMB	0.381	1.84	0.029	2.709	3.693	4.428	3.734	2.756	2.035	1.502	2.129	0.35
PSEN1	0.219	0.222	0.37	−0.728	0.213	−0.337	0.099	−0.045	−0.217	−0.012	−0.533	0.208
PSEN2	−0.133	0.705	−0.852	−0.237	0.334	−0.223	−0.201	0.038	−0.648	0.063	−0.116	0.092
PSENEN	0.975	1.021	0.676	0.126	−0.12	0.028	0.569	0.56	0.297	−0.026	0.081	1.001
PTCRA	−0.057	1.423	0.605	−1.511	1.729	3.145	3.501	−2.559	−3.326	0.59	−1.362	2.093
RFNG	0.62	0.011	−0.652	0.101	−0.491	0.469	0.713	0.508	0.254	0.367	0.19	0.186
**Signal Transductors**												
ATXN1	0.457	−0.280	0.778	−0.286	−0.595	−0.318	−0.497	−0.236	−0.222	−0.104	−0.287	−0.183
ATXN1L	−0.054	−0.014	0.484	0.106	0.277	0.289	0.309	−0.084	−0.054	−0.169	0.080	−0.365
CIR1	−0.118	−0.141	−0.672	0.169	−0.396	0.276	0.084	−0.16	−0.342	−0.142	0.028	−1.056
CREBBP	−0.477	−0.103	−0.352	−0.172	0.561	0.192	−0.171	−0.109	−0.188	−0.12	−0.034	−0.469
CTBP1	0.298	0.19	−0.147	0.105	0.366	0.277	0.334	0.267	−0.067	0.247	0.162	0.32
CTBP2	−0.22	0.402	−0.299	0.133	−0.498	0.208	0.185	0.423	−0.048	0.104	0.175	0.269
EP300	−0.359	−0.233	−0.162	−0.287	0.434	−0.142	−0.675	−0.241	−0.046	−0.127	−0.112	0.017
HDAC1	0.284	0.362	1.072	−0.038	−1.077	−0.103	−0.417	0.705	1.143	0.238	0.068	0.234
HDAC2	0.374	0.377	0.537	0.793	−0.773	−0.198	−0.06	0.427	1.138	0.212	0.75	0.138
KAT2A	1.005	0.042	0.715	1.423	−0.694	0.791	1.224	1.653	1.543	0.952	1.584	0.247
KAT2B	−1.085	−1.399	−1.096	−1.641	−0.941	−0.647	−1.195	−1.306	−1.797	−0.472	−2.002	−1.227
MAML1	−0.099	−0.01	−0.092	−0.02	0.007	0.382	−0.234	0.105	−0.058	0.119	0.163	−0.185
MAML2	−0.963	−2.538	−0.503	−0.441	−2.608	−0.066	−1.309	−0.872	−1.297	−1.363	−0.316	−0.114
MAML3	−0.549	−0.55	−1.127	−0.615	1.093	0.164	−0.67	−0.51	−0.92	0.357	−0.37	−1.142
NCOR2	−0.239	0.034	−0.202	0.175	1.381	0.974	−0.209	−0.33	−0.753	0.005	0.22	0.203
RBPJL	0	0	0	0	0	0	0	0	0	0	0	−9.631
RBPJ	−0.200	0	0	0.011	0.227	0.100	−0.017	−0.016	−0.185	−0.135	−0.025	0
SNW1	−0.004	0.06	0.053	0.128	0.028	0.065	0.002	0.046	0.228	−0.004	0.097	−0.663
**Transcription Factors**												
HES1	0.545	−0.168	1.389	0.004	−2.337	−0.419	−0.418	0.518	1.004	−0.076	0.098	0.666
HES5	0.723	−0.280	1.257	−2.433	1.843	3.421	0.848	−0.503	0.483	−0.708	−1.516	0.110
HEY1	0.398	−0.984	−0.295	−0.669	1.936	1.570	−1.436	−1353	0.541	−0.481	−0.387	0.599
HEY2	−0.310	−0.602	0.352	−0.053	−0.359	0.387	0.045	−0.413	−0.191	0.260	−0.205	−0.006
HEYL	−0.079	0.257	0.096	0.247	−0.127	0.599	−0.476	−0.288	−0.498	0.126	0.170	−0.648
PTCRA	−0.057	1.423	0.605	−1.511	1.729	3.145	3.501	−2.559	−3.326	0.590	−1.362	2.093

**Table 2:** Prognostic effect of the chosen Notch pathway members on DFS in the analyzed tumors. Value represents HR with statistical significance and the color indicates expression level correlated with favorable prognosis: red—higher expression favorable, blue—lower expression favorable (level of the expression is considered relative to the determined cutpoint).

Disease Free Survival													
GENE	BLCA	BRCA	CESC	COAD	KICH	KIRC	KIRP	LUAD	LUSC	OV	PRAD	READ	UCEC
**Ligands and Receptors**													
DLL1	1.73 *			2.44 ***		0.435 *	2.62 *				0.538 **		0.396 *
DLL3	1.91 *	0.5 *	2.18 *				2.76 *						3.15 **
DLL4	0.428 **	4.56 *					2.9 *	2.91 *	1.95 *		1.75 **	0.366 *	
JAG1			3.23 **	0.26 ***			6.32 *				2.13 ***		
JAG2				0.28 ***				4.53 **		0.675 *	1.95 **		0.19 ***
NOTCH1		10.6 **							2.24 *		2 **		0.376 **
NOTCH2										1.37 *	1.73 *		
NOTCH3				0.37 ***			3.11 **				2.75 ***		2.71 **
NOTCH4	0.579 *	1.89 *	2.6 *	0.428 **			2.77 *		2.02 *		1.93 **	0.19 ***	
**Modulators**													
ADAM17	1.99 **			0.523 *	7.79 *		5.13 ***				2.08 **	0.323 *	3.3 ***
APH1A		0.418 *	2.86 *	3.16 ***				0.38 ***			2.04 ***		2.44 *
APH1B			0.398 *										2.05 **
LFNG										0.681 *	0.482 ***	0.146 *	0.325 **
MFNG	0.488 **	0.353 *	0.215 **	2.76 ***						1.63 **	1.99**		0.347 *
NCSTN			2.25*	2.27 **							0.551 **		
NUMB	2.33 **			3.49 ***		0.445 *						5.36 **	
NUMBL	1.79 *	0.531 *	3.88 ***			2.77 ***	2.53 *				1.6 *		2.91 *
PSEN1	1.89 *	0.466 *		3.9 ***		0.402 **				1.4 *		2.73 *	0.368 **
PSEN2				2.76 ***		2.5 **	<100 *			0.632 **	0.565 *	5.13 **	
PSENEN					0.162 *	2.53 **					0.532 **		
RFNG	0.564 *	0.341 *		1.99 *		1.97 *	0.391 *	0.287 **			0.558 **		0.412 *
**Signal Transductors**													
KAT2A	0.527 *	<0.001 *		0.35 ***									
KAT2B			0.426 *	3.5 ***					0.397 *				
MAML1		4.29 *						2.11 *			1.96 **		2.11 *
MAML2	2.08 **	0.143 *		2.05 **		0.182 **			2.37 **		1.7 *		
MAML3		2.13 *	100> *	2.19 **	0.115 *			3.28 **			0.521 **		
NCOR2		3.4 *	0.323 **					2.11 *	3.89 ***				2.23 *
RBPJ		2.97 ***				0.362 **	0.366 *			0.71 *	0.494 *	0.263 **	
RBPJL						0.223 *					1.69 *		2.72 **
**Transcription Factors**													
HES1		0.408 **	2.2 *			2.04 *		0.384 *	0.488 *				0.434 *
HES5	8.94 **	2.47 **		2.38 **	0.113 *		3.58 **	2.06 *					
HEY1				1.99 **			3.33 **	3.17 *		0.665 *	1.93 *	3.5 *	
HEY2					7.55 **					0.64 **			0.458 *
HEYL		3.51 *	3.11 *	0.38 ***			5.13 ***				1.95 **	0.289 *	
PTCRA	0.478 **	2.7 **	0.337 *	2.51 **	>100 *	2.13 *	0.21 ***	0.402 **		0.71 *			

* *p*-value < 0.05; ** *p*-value < 0.01; *** *p*-value < 0.001.

**Table 3:** Prognostic effect of the chosen Notch pathway members on OS in the analyzed tumors. Value represents HR with statistical significance, and the color indicates expression level correlated with favorable prognosis: red—higher expression favorable, blue—lower expression favorable (level of the expression is considered relative to the determined cutpoint).

Overall Survival													
GENE	BLCA	BRCA	CESC	COAD	KICH	KIRC	KIRP	LUAD	LUSC	OV	PRAD	READ	UCEC
**Ligands and Modulators**													
DLL1		0.516 **	2.25 *	2.95 **		0.605 **				1.69 **			
DLL3		2.46 ***	2.62 *	2.73 *		1.84 ***	4.47 ***		0.664 *				2.29 *
DLL4	1.59 *		2.79 ***		0.148 **	0.643 *	8.21 *		1.92 ***	0.71 *			
JAG1			1.73 *		0.243 *	0.553 ***	3.32 *	1.79 **	1.58 *			15.4 **	
JAG2		0.384 ***			0.142 **	0.614 **		2.36 **					
NOTCH1		1.53 *		0.343 *	0.263 *	0.651 *	2.58 **		1.56 *		0.138 **	5.28 *	2.22 *
NOTCH2	1.62 *								1.63 *	1.43 *		0.109 *	2.42 *
NOTCH3	1.68 **	0.656 *			0.227 *	0.518 ***	4.3 ***						2.6 **
NOTCH4		0.668 *	1.92 *		0.098 ***	0.466 ***	3.33 *		1.54 **				
**Modulators**													
ADAM17	2.27 ***		2.22 **			2.56 ***	10.9 ***	1.6 *				0.112 *	
APH1A		0.594 *		0.494 *		0.597 **	2.58 **	0.686 *	0.57 **	0.654 **	0.112 *		2.53 *
APH1B	1.56 *		2.44 ***			0.698 *	0.429 *			1.43 *			
LFNG	0.621 *	0.656 *				1.5 **	0.406 *					14.3 *	
MFNG			0.552 *		0.229 *			0.625 **	1.54 *				0.32 ***
NCSTN	1.6 *	1.66 *	3.29 **		0.172 **	2.03 **	3.14 ***				0.243 *		
NUMB	0.606 *						0.407 *	2.32 *	1.51 *	1.55 **			0.27 ***
NUMBL	1.77 **	0.527 **	0.253 *			3.34 ***	5.67 ***	1.52 *	0.615 *		6.49 *		
PSEN1			1.88 *			0.474 ***		1.52 *	0.622 *		5.52 *		0.264 *
PSEN2		0.489 ***	1.89 *			1.85 ***	2.46 *			0.407 **	<0.001 ***		
PSENEN		0.311 *		3 **		1.94 ***	2.4 *						0.411 *
RFNG		0.221 **						0.633 *		0.633 *			
**Signal Transductors**													
KAT2A		0.276 *			8.03 **	2.54 ***					4.33 *		
KAT2B	0.682 *	2.27 **	0.524 *	0.36 **	4.16 *	0.463 ***		0.636 *	2.34 **				
MAML1	0.682 *			0.472 *			2.44 *					0.143 *	
MAML2						0.405 ***		1.74 *	1.59 *			0.06 ***	
MAML3	2.73 *		1.69 *			0.485 ***			1.45 *				2.94 **
NCOR2		0.458 ***	2.34 **		0.268 *	1.65 **			2.3 **				0.426 *
RBPJ	0.577 *		0.489 *		7.07 **	1.9 ***		0.484 **	1.49 *				0.483 *
RBPJL					7.59 ***	2.29 ***							3.13 **
**Transcription Factors**													
HES1	0.494 ***	0.272 *			0.0863 **						<100 *	4.87 *	
HES5		1.81 **	0.525 *									>100 ***	
HEY1		0.621 *		2.07 *		0.471 ***	2.26 *		0.697 *		0.117 **	4.4 2*	
HEY2	1.84 *	0.56 *			0.156 **	0.604 **				0.723 *		0.18 **	
HEYL	1.89 ***			2 *			3.11 ***		1.41 *				
PTCRA	0.536 *		0.506 *			1.56 **	0.325 **	0.653 *				0.13 *	

* *p*-value < 0.05; ** *p*-value < 0.01; *** *p*-value < 0.001.

**Table 4:** Frequency of mutation and CNVs (mut/CNV) in core components of Notch. Table contains only those members for which values were found in cBioPortal.

NAME	BLCA	BRCA	CESC	COAD	KICH	KIRC	KIRP	LUAD	LUSC	OV	PRAD	READ	UCEC
APH1A	0.4/9.3	-/-	1.0/3.1	-/-	-/-	-/-	-/-	-/-	-/6	0.3/10.2	-/1.4	-/-	-/7.1
CREBBP	6.8/3.8	1.8/4.9	7.2/1.7	9/0.3	-/-	1.1/0.2	2.8/0.7	3.9/1.2	8.4/1.6	2.2/4.2	1.4/1.0	9/0.3	8.9/0.9
DTX1	1.7/1.3	0.6/0.2	0.5/0.3	1.8/-	-/-	0.9/-	-/0.3	1.3/1	-/0.2	0.3/2.2	1.0/1.02	1.8/-	2.8/1.1
EP300	8.9/0.15	1.6/0.2	10.8/2.4	5.4/0.2	-/-	4/-	1.8/0.6	0.9/0.8	4.5/0.8	0.3/2.5	1.2/0.2	5.4/0.2	8.9/1.7
HDAC1	0.4/0.15	-/-	-/-	0.9/0.5	-/-	0.4/-	-/0.3	-/0.8	1.1/0.2	-/-	-/-	0.9/0.5	-/-
HEY1	-/5.5	0.2/9.8	0.5/1.0	1.3/4.1	-/3	0.2/1	0.4/-	0.4/6.4	0.6/3.4	-/8.6	-/6.7	1.3/4.1	-/2.8
MAML2	2.1/2.5	1.2/2.0	2.6/4.4	1.3/0.7	-/-	0.4/1	1.1/1	0.9/1.9	3.9/1.6	0.9/8.1	0.4/1.4	1.3/0.7	3.2/1.5
NCOR2	4.3/-	1.0/1.8	4.1/-	0.9/-	-/-	1.1/-	0.7/0.2	3.5/1.2	3.4/0.2	0.3/3.5	1.4/3.0	0.9/-	6.0/2.0
NCSTN	-/-	0.2/10.7	1.0/3.1	2.2/0.5	-/-	0.2/0.6	0.4/0.3	2.6/10.5	2.2/5.6	-/5	0.4/0.8	2.2/0.5	3.2/4.6
NOTCH1	2.6/2.1	0.6/1.2	5.7/1.0	-/0.11	3/1.5	1.6/0.6	0.7/0.6	4.3/1	7.8/1.2	1.3/4.5	0.8/1.2	-/1.1	3.2/2.4
NOTCH2	3.4/8.1	2/12.10	3.6/2.7	5.4/0.11	3/-	2.7/0.4	0.4/-	2.6/13.6	5.6/8.8	1.3/11.0	2.2/1.0	5.4/1.1	5.6/6.1
NOTCH3	1.3/-	1.0/2.0	4.1/2.1	6.3/0.8	1.5/-	2.4/-	1.4/-	1.3/0.2	3.9/2.4	0.9/16.6	0.8/0.6	6.3/0.8	6.5/7.2
NOTCH4	1.7/2.6	1.0/1.0	6.0/2.4	2.7/0.6	1.5/-	1.3/-	0.4/0.7	10.4/2.3	2.2/0.8	1.6/6.4	0.6/0.6	2.7/0.6	4.8/1.9

## Data Availability

Publicly available datasets were analysed in this study. These data can be found here: TCGA http://gdac.broadinstitute.org/ (17 December 2020). Our data can be found here: https://github.com/dorotaanusewicz/notch_global (17 December 2020).

## References

[1] Andersson E.R., Sandberg R., Lendahl U. (2011). Notch Signaling: Simplicity in Design, Versatility in Function. Development.

[2] Brou C., Logeat F., Gupta N., Bessia C., LeBail O., Doedens J.R., Cumano A., Roux P., Black R.A., Israël A. (2000). A Novel Proteolytic Cleavage Involved in Notch Signaling: The Role of the Disintegrin-Metalloprotease TACE. Mol. Cell.

[3] Kopan R., Ilagan M.X.G. (2009). The Canonical Notch Signaling Pathway: Unfolding the Activation Mechanism. Cell.

[4] Guruharsha K.G., Kankel M.W., Artavanis-Tsakonas S. (2012). The Notch Signalling System: Recent Insights into the Complexity of a Conserved Pathway. Nat. Rev. Genet..

[5] Siebel C., Lendahl U. (2017). Notch Signaling in Development, Tissue Homeostasis, and Disease. Physiol. Rev..

[6] Hogan B.L.M., Barkauskas C.E., Chapman H.A., Epstein J.A., Jain R., Hsia C.C.W., Niklason L., Calle E., Le A., Randell S.H. (2014). Repair and Regeneration of the Respiratory System: Complexity, Plasticity, and Mechanisms of Lung Stem Cell Function. Cell Stem Cell.

[7] Kong Y., Glickman J., Subramaniam M., Shahsafaei A., Allamneni K.P., Aster J.C., Sklar J., Sunday M.E. (2004). Functional Diversity of Notch Family Genes in Fetal Lung Development. Am. J. Physiol. Lung Cell Mol. Physiol..

[8] Hussain M., Xu C., Ahmad M., Yang Y., Lu M., Wu X., Tang L., Wu X. (2017). Notch Signaling: Linking Embryonic Lung Development and Asthmatic Airway Remodeling. Mol. Pharmacol..

[9] Gomi K., Staudt M.R., Salit J., Kaner R.J., Heldrich J., Rogalski A.M., Arbelaez V., Crystal R.G., Walters M.S. (2016). JAG1-Mediated Notch Signaling Regulates Secretory Cell Differentiation of the Human Airway Epithelium. Stem Cell Rev. Rep..

[10] Gomi K., Arbelaez V., Crystal R.G., Walters M.S. (2015). Activation of NOTCH1 or NOTCH3 Signaling Skews Human Airway Basal Cell Differentiation toward a Secretory Pathway. PLoS ONE.

[11] Morimoto M., Nishinakamura R., Saga Y., Kopan R. (2012). Different Assemblies of Notch Receptors Coordinate the Distribution of the Major Bronchial Clara, Ciliated and Neuroendocrine Cells. Development.

[12] Chen L., Al-Awqati Q. (2005). Segmental Expression of Notch and Hairy Genes in Nephrogenesis. Am. J. Physiol. Ren. Physiol..

[13] Chung E., Deacon P., Marable S., Shin J., Park J.-S. (2016). Notch Signaling Promotes Nephrogenesis by Downregulating Six2. Development.

[14] Piscione T.D., Wu M.Y.J., Quaggin S.E. (2004). Expression of Hairy/Enhancer of Split Genes, Hes1 and Hes5, during Murine Nephron Morphogenesis. Gene Expr. Patterns.

[15] Leimeister C., Schumacher N., Gessler M. (2003). Expression of Notch Pathway Genes in the Embryonic Mouse Metanephros Suggests a Role in Proximal Tubule Development. Gene Expr. Patterns.

[16] Cheng H.-T., Kim M., Valerius M.T., Surendran K., Schuster-Gossler K., Gossler A., McMahon A.P., Kopan R. (2007). Notch2, but Not Notch1, Is Required for Proximal Fate Acquisition in the Mammalian Nephron. Development.

[17] Liu Z., Chen S., Boyle S., Zhu Y., Zhang A., Piwnica-Worms D.R., Ilagan M.X.G., Kopan R. (2013). The Extracellular Domain of Notch2 Increases Its Cell-Surface Abundance and Ligand Responsiveness during Kidney Development. Dev. Cell.

[18] Schröder N., Gossler A. (2002). Expression of Notch Pathway Components in Fetal and Adult Mouse Small Intestine. Gene Expr. Patterns.

[19] Sander G.R., Powell B.C. (2004). Expression of Notch Receptors and Ligands in the Adult Gut. J. Histochem. Cytochem..

[20] Akiyama J., Okamoto R., Iwasaki M., Zheng X., Yui S., Tsuchiya K., Nakamura T., Watanabe M. (2010). Delta-like 1 Expression Promotes Goblet Cell Differentiation in Notch-Inactivated Human Colonic Epithelial Cells. Biochem. Biophys. Res. Commun..

[21] Guilmeau S., Flandez M., Mariadason J.M., Augenlicht L.H. (2010). Heterogeneity of Jagged1 Expression in Human and Mouse Intestinal Tumors: Implications for Targeting Notch Signaling. Oncogene.

[22] Abate-Shen C. (2000). Molecular Genetics of Prostate Cancer. Genes Dev..

[23] Robinson E.J., Neal D.E., Collins A.T. (1998). Basal Cells Are Progenitors of Luminal Cells in Primary Cultures of Differentiating Human Prostatic Epithelium. Prostate.

[24] Carvalho F.L.F., Simons B.W., Eberhart C.G., Berman D.M. (2014). Notch Signaling in Prostate Cancer: A Moving Target: Notch Signaling in Prostate Cancer. Prostate.

[25] Leong K.G., Gao W.-Q. (2008). The Notch Pathway in Prostate Development and Cancer. Differentiation.

[26] Frank S.B., Berger P.L., Ljungman M., Miranti C.K. (2017). Human Prostate Luminal Cell Differentiation Requires NOTCH3 Induction by P38-MAPK and MYC. J. Cell Sci..

[27] Wang X.-D., Leow C.C., Zha J., Tang Z., Modrusan Z., Radtke F., Aguet M., de Sauvage F.J., Gao W.-Q. (2006). Notch Signaling Is Required for Normal Prostatic Epithelial Cell Proliferation and Differentiation. Dev. Biol..

[28] Belandia B., Powell S.M., García-Pedrero J.M., Walker M.M., Bevan C.L., Parker M.G. (2005). Hey1, a Mediator of Notch Signaling, Is an Androgen Receptor Corepressor. Mol. Cell. Biol..

[29] Fiúza U.-M., Arias A.M. (2007). Cell and Molecular Biology of Notch. J. Endocrinol..

[30] Vanorny D.A., Mayo K.E. (2017). The Role of Notch Signaling in the Mammalian Ovary. Reproduction.

[31] Raafat A., Goldhar A.S., Klauzinska M., Xu K., Amirjazil I., McCurdy D., Lashin K., Salomon D., Vonderhaar B.K., Egan S. (2011). Expression of Notch Receptors, Ligands, and Target Genes during Development of the Mouse Mammary Gland. J. Cell Physiol..

[32] Dontu G., Jackson K.W., McNicholas E., Kawamura M.J., Abdallah W.M., Wicha M.S. (2004). Role of Notch Signaling in Cell-Fate Determination of Human Mammary Stem/Progenitor Cells. Breast Cancer Res..

[33] Aithal M.G.S., Rajeswari N. (2013). Role of Notch Signalling Pathway in Cancer and Its Association with DNA Methylation. J. Genet..

[34] Previs R.A., Coleman R.L., Harris A.L., Sood A.K. (2015). Molecular Pathways: Translational and Therapeutic Implications of the Notch Signaling Pathway in Cancer. Clin. Cancer Res..

[35] Lobry C., Oh P., Aifantis I. (2011). Oncogenic and Tumor Suppressor Functions of Notch in Cancer: It’s NOTCH What You Think. J. Exp. Med..

[36] Aster J.C., Pear W.S., Blacklow S.C. (2017). The Varied Roles of Notch in Cancer. Annu. Rev. Pathol..

[37] Wei L., Jin Z., Yang S., Xu Y., Zhu Y., Ji Y. (2018). TCGA-Assembler 2: Software Pipeline for Retrieval and Processing of TCGA/CPTAC Data. Bioinformatics.

[38] Liberzon A., Birger C., Thorvaldsdóttir H., Ghandi M., Mesirov J.P., Tamayo P. (2015). The Molecular Signatures Database (MSigDB) Hallmark Gene Set Collection. Cell Syst..

[39] Yevshin I., Sharipov R., Kolmykov S., Kondrakhin Y., Kolpakov F. (2019). GTRD: A Database on Gene Transcription Regulation-2019 Update. Nucleic Acids Res..

[40] Diaz-Papkovich A., Anderson-Trocmé L., Ben-Eghan C., Gravel S. (2019). UMAP Reveals Cryptic Population Structure and Phenotype Heterogeneity in Large Genomic Cohorts. PLoS Genet..

[41] Ogłuszka M., Orzechowska M., Jędroszka D., Witas P., Bednarek A.K. (2019). Evaluate Cutpoints: Adaptable Continuous Data Distribution System for Determining Survival in Kaplan-Meier Estimator. Comput. Methods Programs Biomed..

[42] Cerami E., Gao J., Dogrusoz U., Gross B.E., Sumer S.O., Aksoy B.A., Jacobsen A., Byrne C.J., Heuer M.L., Larsson E. (2012). The CBio Cancer Genomics Portal: An Open Platform for Exploring Multidimensional Cancer Genomics Data. Cancer Discov..

[43] Donnem T., Andersen S., Al-Shibli K., Al-Saad S., Busund L.-T., Bremnes R.M. (2010). Prognostic Impact of Notch Ligands and Receptors in Nonsmall Cell Lung Cancer: Coexpression of Notch-1 and Vascular Endothelial Growth Factor-A Predicts Poor Survival. Cancer.

[44] Cai H., Lu W., Zhang Y., Liu H., Wang Z., Shen Y. (2019). Specific Inhibition of Notch1 Signaling Suppresses Properties of Lung Cancer Stem Cells. J. Cancer Res. Ther..

[45] Deng F., Yang Z.-F., Sun C.-Q. (2017). The Role of Notch1 Genes in Lung Cancer A594 Cells and the Impact on Chemosensitivity. Eur. Rev. Med. Pharmacol. Sci..

[46] Baumgart A., Seidl S., Vlachou P., Michel L., Mitova N., Schatz N., Specht K., Koch I., Schuster T., Grundler R. (2010). ADAM17 Regulates Epidermal Growth Factor Receptor Expression through the Activation of Notch1 in Non–Small Cell Lung Cancer. Cancer Res..

[47] Wael H., Yoshida R., Kudoh S., Hasegawa K., Niimori-Kita K., Ito T. (2014). Notch1 Signaling Controls Cell Proliferation, Apoptosis and Differentiation in Lung Carcinoma. Lung Cancer.

[48] Liu Z.-Y., Wu T., Li Q., Wang M.-C., Jing L., Ruan Z.-P., Yao Y., Nan K.-J., Guo H. (2016). Notch Signaling Components: Diverging Prognostic Indicators in Lung Adenocarcinoma. Medicine.

[49] Chang W.-H., Ho B.-C., Hsiao Y.-J., Chen J.-S., Yeh C.-H., Chen H.-Y., Chang G.-C., Su K.-Y., Yu S.-L. (2016). JAG1 Is Associated with Poor Survival through Inducing Metastasis in Lung Cancer. PLoS ONE.

[50] Ye Y., Zhang Z., Fan X., Xu X., Chen M., Chang B., Zhang Y. (2013). Notch3 Overexpression Associates with Poor Prognosis in Human Non-Small-Cell Lung Cancer. Med. Oncol..

[51] Shi C., Qian J., Ma M., Zhang Y., Han B. (2014). Notch 3 Protein, Not Its Gene Polymorphism, Is Associated with the Chemotherapy Response and Prognosis of Advanced NSCLC Patients. Cell. Physiol. Biochem..

[52] Hassan W.A., Yoshida R., Kudoh S., Motooka Y., Ito T. (2016). Evaluation of Role of Notch3 Signaling Pathway in Human Lung Cancer Cells. J. Cancer Res. Clin. Oncol..

[53] Chu D., Zhang Z., Zhou Y., Wang W., Li Y., Zhang H., Dong G., Zhao Q., Ji G. (2011). Notch1 and Notch2 Have Opposite Prognostic Effects on Patients with Colorectal Cancer. Ann. Oncol..

[54] Fender A.W., Nutter J.M., Fitzgerald T.L., Bertrand F.E., Sigounas G. (2015). Notch-1 Promotes Stemness and Epithelial to Mesenchymal Transition in Colorectal Cancer. J. Cell. Biochem..

[55] Sugiyama M., Oki E., Nakaji Y., Tsutsumi S., Ono N., Nakanishi R., Sugiyama M., Nakashima Y., Sonoda H., Ohgaki K. (2016). High Expression of the Notch Ligand Jagged-1 Is Associated with Poor Prognosis after Surgery for Colorectal Cancer. Cancer Sci..

[56] Lu J., Ye X., Fan F., Xia L., Bhattacharya R., Bellister S., Tozzi F., Sceusi E., Zhou Y., Tachibana I. (2013). Endothelial Cells Promote the Colorectal Cancer Stem Cell Phenotype through a Soluble Form of Jagged-1. Cancer Cell.

[57] Pelullo M., Nardozza F., Zema S., Quaranta R., Nicoletti C., Besharat Z.M., Felli M.P., Cerbelli B., d’Amati G., Palermo R. (2019). Kras/ADAM17-Dependent Jag1-ICD Reverse Signaling Sustains Colorectal Cancer Progression and Chemoresistance. Cancer Res..

[58] Reedijk M., Odorcic S., Zhang H., Chetty R., Tennert C., Dickson B.C., Lockwood G., Gallinger S., Egan S.E. (2008). Activation of Notch Signaling in Human Colon Adenocarcinoma. Int. J. Oncol..

[59] Vaish V., Kim J., Shim M. (2017). Jagged-2 (JAG2) Enhances Tumorigenicity and Chemoresistance of Colorectal Cancer Cells. Oncotarget.

[60] Serafin V., Persano L., Moserle L., Esposito G., Ghisi M., Curtarello M., Bonanno L., Masiero M., Ribatti D., Stürzl M. (2011). Notch3 Signalling Promotes Tumour Growth in Colorectal Cancer. J. Pathol..

[61] Pasto A., Serafin V., Pilotto G., Lago C., Bellio C., Trusolino L., Bertotti A., Hoey T., Plateroti M., Esposito G. (2014). NOTCH3 Signaling Regulates MUSASHI-1 Expression in Metastatic Colorectal Cancer Cells. Cancer Res..

[62] Ozawa T., Kazama S., Akiyoshi T., Murono K., Yoneyama S., Tanaka T., Tanaka J., Kiyomatsu T., Kawai K., Nozawa H. (2014). Nuclear Notch3 Expression Is Associated with Tumor Recurrence in Patients with Stage II and III Colorectal Cancer. Ann. Surg. Oncol..

[63] Jędroszka D., Orzechowska M., Bednarek A.K. (2017). Predictive Values of Notch Signalling in Renal Carcinoma. Arch. Med. Sci..

[64] Zhuang Z., Lin J., Huang Y., Lin T., Zheng Z., Ma X. (2017). Notch 1 Is a Valuable Therapeutic Target against Cell Survival and Proliferation in Clear Cell Renal Cell Carcinoma. Oncol. Lett..

[65] Ai Q., Ma X., Huang Q., Liu S., Shi T., Zhang C., Zhu M., Zhang Y., Wang B., Ni D. (2012). High-Level Expression of Notch1 Increased the Risk of Metastasis in T1 Stage Clear Cell Renal Cell Carcinoma. PLoS ONE.

[66] Lee J.N., Chun S.Y., Lee H.J., Ha Y.-S., Kim H.T., Yoo E.S., Kwon T.G., Kim T.-H. (2016). High Notch1 Expression Correlates with Tumor Stage and Size in Clear Cell Renal Cell Carcinoma. Korean J. Urol. Oncol..

[67] Wu K., Xu L., Zhang L., Lin Z., Hou J. (2011). High Jagged1 Expression Predicts Poor Outcome in Clear Cell Renal Cell Carcinoma. Jpn. J. Clin. Oncol..

[68] Hu G.-H., Liu H., Lai P., Guo Z.-F., Xu L., Yao X.-D., Zheng J.-H., Liu M., Xu Y.-F. (2014). Delta-like Ligand 4 (Dll4) Predicts the Prognosis of Clear Cell Renal Cell Carcinoma, and Anti-Dll4 Suppresses Tumor Growth in Vivo. Int. J. Clin. Exp. Pathol..

[69] Huang Q.B., Ma X., Li H.Z., Ai Q., Liu S.W., Zhang Y., Gao Y., Fan Y., Ni D., Wang B.J. (2014). Endothelial Delta-like 4 (DLL4) Promotes Renal Cell Carcinoma Hematogenous Metastasis. Oncotarget.

[70] Sun S., Du R., Gao J., Ning X., Xie H., Lin X., Liu J., Fan D. (2009). Expression and Clinical Significance of Notch Receptors in Human Renal Cell Carcinoma. Pathology.

[71] Cairns P. (2010). Renal Cell Carcinoma. Cancer Biomark..

[72] Maraver A., Fernandez-Marcos P.J., Cash T.P., Mendez-Pertuz M., Dueñas M., Maietta P., Martinelli P., Muñoz-Martin M., Martínez-Fernández M., Cañamero M. (2015). NOTCH Pathway Inactivation Promotes Bladder Cancer Progression. J. Clin. Investig..

[73] Rampias T., Vgenopoulou P., Avgeris M., Polyzos A., Stravodimos K., Valavanis C., Scorilas A., Klinakis A. (2014). A New Tumor Suppressor Role for the Notch Pathway in Bladder Cancer. Nat. Med..

[74] Greife A., Jankowiak S., Steinbring J., Nikpour P., Niegisch G., Hoffmann M.J., Schulz W.A. (2014). Canonical Notch Signalling Is Inactive in Urothelial Carcinoma. BMC Cancer.

[75] Hayashi T., Gust K.M., Wyatt A.W., Goriki A., Jäger W., Awrey S., Li N., Oo H.Z., Altamirano-Dimas M., Buttyan R. (2016). Not All NOTCH Is Created Equal: The Oncogenic Role of NOTCH2 in Bladder Cancer and Its Implications for Targeted Therapy. Clin. Cancer Res..

[76] Wu X., Chen B., Shi H., Zhou J., Zhou F., Cao J., Sun X. (2019). MiR-758-3p Suppresses Human Bladder Cancer Cell Proliferation, Migration and Invasion by Targeting NOTCH2. Exp. Ther. Med..

[77] Zhang H., Liu L., Liu C., Pan J., Lu G., Zhou Z., Chen Z., Qian C. (2017). Notch3 Overexpression Enhances Progression and Chemoresistance of Urothelial Carcinoma. Oncotarget.

[78] Patel N.S. (2006). Up-Regulation of Endothelial Delta-like 4 Expression Correlates with Vessel Maturation in Bladder Cancer. Clin. Cancer Res..

[79] Koshkin V.S., Garcia J.A., Reynolds J., Elson P., Magi-Galluzzi C., McKenney J.K., Isse K., Bishop E., Saunders L.R., Balyimez A. (2019). Transcriptomic and Protein Analysis of Small-Cell Bladder Cancer (SCBC) Identifies Prognostic Biomarkers and DLL3 as a Relevant Therapeutic Target. Clin. Cancer Res..

[80] Wang X.-D., Shou J., Wong P., French D.M., Gao W.-Q. (2004). Notch1-Expressing Cells Are Indispensable for Prostatic Branching Morphogenesis during Development and Re-Growth Following Castration and Androgen Replacement. J. Biol. Chem..

[81] Rice M.A., Hsu E.-C., Aslan M., Ghoochani A., Su A., Stoyanova T. (2019). Loss of Notch1 Activity Inhibits Prostate Cancer Growth and Metastasis and Sensitizes Prostate Cancer Cells to Antiandrogen Therapies. Mol. Cancer Ther..

[82] Stoyanova T., Riedinger M., Lin S., Faltermeier C.M., Smith B.A., Zhang K.X., Going C.C., Goldstein A.S., Lee J.K., Drake J.M. (2016). Activation of Notch1 Synergizes with Multiple Pathways in Promoting Castration-Resistant Prostate Cancer. Proc. Natl. Acad. Sci. USA.

[83] Zhang L., Sha J., Yang G., Huang X., Bo J., Huang Y. (2017). Activation of Notch Pathway Is Linked with Epithelial-Mesenchymal Transition in Prostate Cancer Cells. Cell Cycle.

[84] Zhu H., Zhou X., Redfield S., Lewin J., Miele L. (2013). Elevated Jagged-1 and Notch-1 Expression in High Grade and Metastatic Prostate Cancers. Am. J. Transl. Res..

[85] Kim A.R., Gu M.J. (2019). The Clinicopathologic Significance of Notch3 Expression in Prostate Cancer. Int. J. Clin. Exp. Pathol..

[86] Danza G., Di Serio C., Ambrosio M.R., Sturli N., Lonetto G., Rosati F., Rocca B.J., Ventimiglia G., del Vecchio M.T., Prudovsky I. (2013). Notch3 Is Activated by Chronic Hypoxia and Contributes to the Progression of Human Prostate Cancer: Notch3 and Prostate Cancer. Int. J. Cancer.

[87] Zhang J., Kuang Y., Wang Y., Xu Q., Ren Q. (2017). Notch-4 Silencing Inhibits Prostate Cancer Growth and EMT via the NF-ΚB Pathway. Apoptosis.

[88] Puca L., Gavyert K., Sailer V., Conteduca V., Dardenne E., Sigouros M., Isse K., Kearney M., Vosoughi A., Fernandez L. (2019). Delta-like Protein 3 Expression and Therapeutic Targeting in Neuroendocrine Prostate Cancer. Sci. Transl. Med..

[89] Srivastava S., Ramdass B., Nagarajan S., Rehman M., Mukherjee G., Krishna S. (2010). Notch1 Regulates the Functional Contribution of RhoC to Cervical Carcinoma Progression. Br. J. Cancer.

[90] Yousif N.G., Sadiq A.M., Yousif M.G., Al-Mudhafar R.H., Al-Baghdadi J.J., Hadi N. (2015). Notch1 Ligand Signaling Pathway Activated in Cervical Cancer: Poor Prognosis with High-Level JAG1/Notch1. Arch. Gynecol. Obs..

[91] Talora C., Sgroi D.C., Crum C.P., Dotto G.P. (2002). Specific Down-Modulation of Notch1 Signaling in Cervical Cancer Cells Is Required for Sustained HPV-E6/E7 Expression and Late Steps of Malignant Transformation. Genes Dev..

[92] Talora C., Cialfi S., Segatto O., Morrone S., Kim Choi J., Frati L., Paolo Dotto G., Gulino A., Screpanti I. (2005). Constitutively Active Notch1 Induces Growth Arrest of HPV-Positive Cervical Cancer Cells via Separate Signaling Pathways. Exp. Cell Res..

[93] Mitsuhashi Y., Horiuchi A., Miyamoto T., Kashima H., Suzuki A., Shiozawa T. (2012). Prognostic Significance of Notch Signalling Molecules and Their Involvement in the Invasiveness of Endometrial Carcinoma Cells. Histopathology.

[94] Cobellis L., Caprio F., Trabucco E., Mastrogiacomo A., Coppola G., Manente L., Colacurci N., De Falco M., De Luca A. (2008). The Pattern of Expression of Notch Protein Members in Normal and Pathological Endometrium. J. Anat..

[95] Jonusiene V., Sasnauskiene A., Lachej N., Kanopiene D., Dabkeviciene D., Sasnauskiene S., Kazbariene B., Didziapetriene J. (2013). Down-Regulated Expression of Notch Signaling Molecules in Human Endometrial Cancer. Med. Oncol..

[96] Sasnauskienė A., Jonušienė V., Krikštaponienė A., Butkytė S., Dabkevičienė D., Kanopienė D., Kazbarienė B., Didžiapetrienė J. (2014). NOTCH1, NOTCH3, NOTCH4, and JAG2 Protein Levels in Human Endometrial Cancer. Medicina.

[97] Choi J.-H., Park J.T., Davidson B., Morin P.J., Shih I.-M., Wang T.-L. (2008). Jagged-1 and Notch3 Juxtacrine Loop Regulates Ovarian Tumor Growth and Adhesion. Cancer Res..

[98] Jung S.G., Kwon Y.D., Song J.A., Back M.J., Lee S.Y., Lee C., Hwang Y.Y., An H.J. (2010). Prognostic Significance of Notch 3 Gene Expression in Ovarian Serous Carcinoma. Cancer Sci..

[99] Wang M., Wang J., Wang L., Wu L., Xin X. (2010). Notch1 Expression Correlates with Tumor Differentiation Status in Ovarian Carcinoma. Med. Oncol..

[100] Hu W., Lu C., Dong H.H., Huang J., Shen D., Stone R.L., Nick A.M., Shahzad M.M.K., Mora E., Jennings N.B. (2011). Biological Roles of the Delta Family Notch Ligand Dll4 in Tumor and Endothelial Cells in Ovarian Cancer. Cancer Res..

[101] Chen C., Wang X., Huang S., Wang L., Han L., Yu S. (2017). Prognostic Roles of Notch Receptor MRNA Expression in Human Ovarian Cancer. Oncotarget.

[102] Yuan X., Zhang M., Wu H., Xu H., Han N., Chu Q., Yu S., Chen Y., Wu K. (2015). Expression of Notch1 Correlates with Breast Cancer Progression and Prognosis. PLoS ONE.

[103] Farnie G., Clarke R.B., Spence K., Pinnock N., Brennan K., Anderson N.G., Bundred N.J. (2007). Novel Cell Culture Technique for Primary Ductal Carcinoma in Situ: Role of Notch and Epidermal Growth Factor Receptor Signaling Pathways. J. Natl. Cancer Inst..

[104] Reedijk M., Odorcic S., Chang L., Zhang H., Miller N., McCready D.R., Lockwood G., Egan S.E. (2005). High-Level Coexpression of JAG1 and NOTCH1 Is Observed in Human Breast Cancer and Is Associated with Poor Overall Survival. Cancer Res..

[105] Leong K.G., Niessen K., Kulic I., Raouf A., Eaves C., Pollet I., Karsan A. (2007). Jagged1-Mediated Notch Activation Induces Epithelial-to-Mesenchymal Transition through Slug-Induced Repression of E-Cadherin. J. Exp. Med..

[106] Simões B.M., O’Brien C.S., Eyre R., Silva A., Yu L., Sarmiento-Castro A., Alférez D.G., Spence K., Santiago-Gómez A., Chemi F. (2015). Anti-Estrogen Resistance in Human Breast Tumors Is Driven by JAG1-NOTCH4-Dependent Cancer Stem Cell Activity. Cell Rep..

[107] Weng A.P., Ferrando A.A., Lee W., Morris J.P., Silverman L.B., Sanchez-Irizarry C., Blacklow S.C., Look A.T., Aster J.C. (2004). Activating mutations of NOTCH1 in human T cell acute lymphoblastic leukemia. Science.

[108] Marquez-Exposito L., Cantero-Navarro E., Lavoz C., Fierro-Fernández M., Poveda J., Rayego-Mateos S., Rodrigues-Diez R.R., Morgado-Pascual J.L., Orejudo M., Mezzano S. (2018). Could Notch signaling pathway be a potential therapeutic option in renal diseases?. Nefrologia.

[109] Mukherjee M., Fogarty E., Janga M., Surendran K. (2019). Notch Signaling in Kidney Development, Maintenance, and Disease. Biomolecules.

[110] Izumchenko E., Sun K., Jones S., Brait M., Agrawal N., Koch W., McCord C.L., Riley D.R., Angiuoli S.V., Velculescu V.E. (2015). Notch1 mutations are drivers of oral tumorigenesis. Cancer Prev. Res..

[111] Agrawal N., Frederick M.J., Pickering C.R., Bettegowda C., Chang K., Li R.J., Fakhry C., Xie T.X., Zhang J., Wang J. (2011). Exome sequencing of head and neck squamous cell carcinoma reveals inactivating mutations in NOTCH1. Science.

[112] Kovall R.A., Gebelein B., Sprinzak D., Kopan R. (2017). The Canonical Notch Signaling Pathway: Structural and Biochemical Insights into Shape, Sugar, and Force. Dev. Cell.

[113] Yi F., Amarasinghe B., Dang T.P. (2013). Manic Fringe Inhibits Tumor Growth by Suppressing Notch3 Degradation in Lung Cancer. Am. J. Cancer Res..

[114] Kikuchi H., Sakakibara-Konishi J., Furuta M., Kikuchi E., Kikuchi J., Oizumi S., Hida Y., Kaga K., Kinoshita I., Dosaka-Akita H. (2018). Numb Has Distinct Function in Lung Adenocarcinoma and Squamous Cell Carcinoma. Oncotarget.

[115] Yingjie L., Jian T., Changhai Y., Jingbo L. (2013). Numblike Regulates Proliferation, Apoptosis, and Invasion of Lung Cancer Cell. Tumor Biol..

[116] Sima J., Zhang B., Yu Y., Sima X., Mao Y. (2015). Overexpression of Numb Suppresses Growth, Migration, and Invasion of Human Clear Cell Renal Cell Carcinoma Cells. Tumour Biol..

[117] López-Arribillaga E., Rodilla V., Colomer C., Vert A., Shelton A., Cheng J.H., Yan B., Gonzalez-Perez A., Junttila M.R., Iglesias M. (2018). Manic Fringe Deficiency Imposes Jagged1 Addiction to Intestinal Tumor Cells. Nat. Commun..

[118] García-Heredia J.M., Verdugo Sivianes E.M., Lucena-Cacace A., Molina-Pinelo S., Carnero A. (2016). Numb-like (NumbL) Downregulation Increases Tumorigenicity, Cancer Stem Cell-like Properties and Resistance to Chemotherapy. Oncotarget.

[119] Zhang S., Chung W., Wu G., Egan S.E., Xu K. (2014). Tumor-Suppressive Activity of Lunatic Fringe in Prostate through Differential Modulation of Notch Receptor Activation. Neoplasia.

[120] Lin P., Sun X., Feng T., Zou H., Jiang Y., Liu Z., Zhao D., Yu X. (2012). ADAM17 Regulates Prostate Cancer Cell Proliferation through Mediating Cell Cycle Progression by EGFR/PI3K/AKT Pathway. Mol. Cell. Biochem..

[121] Tosoni D., Pambianco S., Ekalle Soppo B., Zecchini S., Bertalot G., Pruneri G., Viale G., Di Fiore P.P., Pece S. (2017). Pre-Clinical Validation of a Selective Anti-Cancer Stem Cell Therapy for Numb-Deficient Human Breast Cancers. EMBO Mol. Med..

[122] Vázquez-Ulloa E., Ramos-Cruz A.C., Prada D., Avilés-Salas A., Chávez-Blanco A.D., Herrera L.A., Lizano M., Contreras-Paredes A. (2018). Loss of Nuclear NOTCH1, but Not Its Negative Regulator NUMB, Is an Independent Predictor of Cervical Malignancy. Oncotarget.

[123] Rong C., Feng Y., Ye Z. (2017). Notch Is a Critical Regulator in Cervical Cancer by Regulating Numb Splicing. Oncol. Lett..

[124] Kulic I., Robertson G., Chang L., Baker J.H.E., Lockwood W.W., Mok W., Fuller M., Fournier M., Wong N., Chou V. (2015). Loss of the Notch Effector RBPJ Promotes Tumorigenesis. J. Exp. Med..

[125] Alves-Guerra M.-C., Ronchini C., Capobianco A.J. (2007). Mastermind-like 1 Is a Specific Coactivator of Beta-Catenin Transcription Activation and Is Essential for Colon Carcinoma Cell Survival. Cancer Res..

[126] Hansson M.L., Behmer S., Ceder R., Mohammadi S., Preta G., Grafström R.C., Fadeel B., Wallberg A.E. (2012). MAML1 Acts Cooperatively with EGR1 to Activate EGR1-Regulated Promoters: Implications for Nephrogenesis and the Development of Renal Cancer. PLoS ONE.

[127] Shariat Razavi S.M., Forghanifard M.M., Kordi-Tamandani D.M., Abbaszadegan M.R. (2019). MAML1 Regulates EMT Markers Expression through NOTCH-Independent Pathway in Breast Cancer Cell Line MCF7. Biochem. Biophys. Res. Commun..

[128] Chen L., Wei T., Si X., Wang Q., Li Y., Leng Y., Deng A., Chen J., Wang G., Zhu S. (2013). Lysine Acetyltransferase GCN5 Potentiates the Growth of Non-Small Cell Lung Cancer via Promotion of E2F1, Cyclin D1, and Cyclin E1 Expression. J. Biol. Chem..

[129] Yin Y.-W., Jin H.-J., Zhao W., Gao B., Fang J., Wei J., Zhang D.D., Zhang J., Fang D. (2015). The Histone Acetyltransferase GCN5 Expression Is Elevated and Regulated by C-Myc and E2F1 Transcription Factors in Human Colon Cancer. Gene Expr..

[130] Tang Y., Hu C., Yang H., Cao L., Li Y., Deng P., Huang L. (2014). Rnd3 Regulates Lung Cancer Cell Proliferation through Notch Signaling. PLoS ONE.

[131] Liu S., Liu W.-H., Diao Z.-L., Zhang A.-H., Guo W., Han X., Huang H.-D. (2020). LncRNA RP11-567G11.1 Accelerates the Proliferation and Invasion of Renal Cell Carcinoma through Activating the Notch Pathway. Eur. Rev. Med. Pharm. Sci..

[132] Bonyadi Rad E., Hammerlindl H., Wels C., Popper U., Ravindran Menon D., Breiteneder H., Kitzwoegerer M., Hafner C., Herlyn M., Bergler H. (2016). Notch4 Signaling Induces a Mesenchymal-Epithelial-like Transition in Melanoma Cells to Suppress Malignant Behaviors. Cancer Res..

[133] Guo Z., Jin X., Jia H. (2013). Inhibition of ADAM-17 More Effectively down-Regulates the Notch Pathway than That of γ-Secretase in Renal Carcinoma. J. Exp. Clin. Cancer Res..

[134] Yuan R., Ke J., Sun L., He Z., Zou Y., He X., Chen Y., Wu X., Cai Z., Wang L. (2015). HES1 Promotes Metastasis and Predicts Poor Survival in Patients with Colorectal Cancer. Clin. Exp. Metastasis.

[135] Gao F., Zhang Y., Wang S., Liu Y., Zheng L., Yang J., Huang W., Ye Y., Luo W., Xiao D. (2014). Hes1 Is Involved in the Self-Renewal and Tumourigenicity of Stem-like Cancer Cells in Colon Cancer. Sci. Rep..

[136] Weng M., Tsao P., Lin H., Tung C., Change M., Chang Y., Wong J., Wei S. (2015). Hes1 Increases the Invasion Ability of Colorectal Cancer Cells via the STAT3-MMP14 Pathway. PLoS ONE.

[137] Candy P.A., Phillips M.R., Redfern A.D., Colley S.M., Davidson J.A., Stuart L.M., Wood B.A., Zeps N., Leedman P.J. (2013). Notch-Induced Transcription Factors Are Predictive of Survival and 5-Fluorouracil Response in Colorectal Cancer Patients. Br. J. Cancer.

[138] Bolós V., Mira E., Martínez-Poveda B., Luxán G., Cañamero M., Martínez-A C., Mañes S., de la Pompa J.L. (2013). Notch Activation Stimulates Migration of Breast Cancer Cells and Promotes Tumor Growth. Breast Cancer Res..

[139] Wong N.K.Y., Fuller M., Sung S., Wong F., Karsan A. (2012). Heterogeneity of Breast Cancer Stem Cells as Evidenced with Notch-Dependent and Notch-Independent Populations. Cancer Med..

[140] Pratap J., Lian J.B., Javed A., Barnes G.L., van Wijnen A.J., Stein J.L., Stein G.S. (2006). Regulatory Roles of Runx2 in Metastatic Tumor and Cancer Cell Interactions with Bone. Cancer Metastasis Rev..

[141] Liu J., Ye F., Chen H., Lü W., Zhou C., Xie X. (2007). Expression of differentiation associated protein Hes1 and Hes5 in cervical squamous carcinoma and its precursors. Int. J. Gynecol. Cancer.

[142] Hanahan D., Weinberg R.A. (2011). Hallmarks of Cancer: The Next Generation. Cell.

[143] Feitelson M.A., Arzumanyan A., Kulathinal R.J., Blain S.W., Holcombe R.F., Mahajna J., Marino M., Martinez-Chantar M.L., Nawroth R., Sanchez-Garcia I. (2015). Sustained Proliferation in Cancer: Mechanisms and Novel Therapeutic Targets. Semin. Cancer Biol..

[144] Chen F., Zhang Y., Şenbabaoğlu Y., Ciriello G., Yang L., Reznik E., Shuch B., Micevic G., De Velasco G., Shinbrot E. (2016). Multilevel Genomics-Based Taxonomy of Renal Cell Carcinoma. Cell Rep..

[145] Lamouille S., Xu J., Derynck R. (2014). Molecular Mechanisms of Epithelial-Mesenchymal Transition. Nat. Rev. Mol. Cell Biol..

[146] Zhang E., Weinberg R.A. (2018). Epithelial-to-mesenchymal transition in cancer: Complexity and opportunities. Front. Med..

[147] Stefania D.D., Vergara D. (2017). The Many-Faced Program of Epithelial-Mesenchymal Transition: A System Biology-Based View. Front. Oncol..

[148] Relli V., Trerotola M., Guerra E., Alberti S. (2018). Distinct Lung Cancer Subtypes Associate to Distinct Drivers of Tumor Progression. Oncotarget.

[149] Chen M., Liu X., Du J., Wang X.-J., Xia L. (2017). Differentiated Regulation of Immune-Response Related Genes between LUAD and LUSC Subtypes of Lung Cancers. Oncotarget.

